# Supplementary material for: Dynamic patterns of information flow in complex networks
Source: Nat Commun. 2017 Dec 19;8:2181. doi: 10.1038/s41467-017-01916-3 (PMC5736766; doi:10.1038/s41467-017-01916-3)
Supplement: Supplementary file 1 — Supplementary Information [file 41467_2017_1916_MOESM1_ESM.pdf]

Supplementary Note 1

Analytic derivation of ω and ξ

We consider a sparse network with $N \to \infty$ nodes and L links, whose time dependent activities $x_i(t)$, $i = 1, \dots, N$, are driven by the nonlinear dynamic equation [1, 2]

$$\frac{\mathrm{d}x_i}{\mathrm{d}t} = M_0\big(x_i(t)\big) + \sum_{j=1}^{N} A_{ij} M_1\big(x_i(t)\big) M_2\big(x_j(t)\big). \tag{1}$$

In (1) the connectivity is captured by A_{ij}, a weighed and directed network, and the dynamic model $\mathbf{M} = \big(M_0(x), M_1(x), M_2(x)\big)$ is described by the Hahn series [3]

$$M_0(x) = \sum_{n=0}^{\infty} a_n (x - x_0)^{\Gamma_0(n)} \tag{2}$$

$$M_1(x) = \sum_{n=0}^{\infty} b_n (x - x_0)^{\Gamma_1(n)} \tag{3}$$

$$M_2(x) = \sum_{n=0}^{\infty} c_n (x - x_0)^{\Gamma_2(n)}, \tag{4}$$

a generalization of the Taylor expansion, to include both negative and real powers; here $\Gamma_i(n)$ represent a countable set of ordered real powers, namely

$$\Gamma_i(0) < \Gamma_i(1) < \dots . \tag{5}$$

We obtain the fixed point/points of (1) by setting the derivative on the l.h.s. to zero, obtaining

$$M_0(x_i) + \sum_{j=1}^{N} A_{ij} M_1(x_i) M_2(x_j) = 0, \tag{6}$$

where we denote by x_i the steady-state activities, distinguished from the time dependent activities $x_i(t)$.

We track the propagation of signals in the system by introducing a small time-independent perturbation $\mathrm{d}x_j$ on the steady-state activity of a source node j, observing the response of all remaining target nodes $i = 1, \dots, N$. This is achieved by setting an effective boundary condition

$$x_j(t) = x_j + \mathrm{d}x_j \tag{7}$$

on the jth equation of (1). The remaining $N-1$ equations are unchanged, namely (1) transforms to

$$\begin{aligned} &\frac{\mathrm{d}x_i}{\mathrm{d}t} = M_0\big(x_i(t)\big) + \sum_{k=1}^{N} A_{ik} M_1\big(x_i(t)\big) M_2\big(x_k(t)\big) \quad i \neq j \\ &x_j(t) = x_j + \mathrm{d}x_j \end{aligned} . \tag{8}$$

The boundary condition (7) forces the system into a new fixed point, in which all x_i are shifted by $\mathrm{d}x_i$, providing the response matrix [1], which we define in the main text to be

$$G_{ij} = \left| \frac{\mathrm{d}x_i/x_i}{\mathrm{d}x_j/x_j} \right| = \left| \frac{\mathrm{d}\ln x_i}{\mathrm{d}\ln x_j} \right| . \tag{9}$$

To obtain G_{ij} we begin with the local response matrix

$$R_{ij} = \left| \frac{\partial x_i/x_i}{\partial x_j/x_j} \right| , \tag{10}$$

in which the partial derivative (∂) indicates that all other node activities x_k, $k \neq i, j$, are held constant. Hence while G_{ij} (9) captures the response of i's activity to a perturbation in j within the context of an active network, R_{ij} tracks only the direct impact of j on i, in isolation from all other network effects. The two matrices R_{ij} and G_{ij} can be linked through the chain rule for multivariate functions

$$\frac{\mathrm{d}x_i}{\mathrm{d}x_j} = \frac{\partial x_i}{\partial x_j} + \sum_{\substack{k=1 \\ k \neq j}}^{N} \frac{\partial x_i}{\partial x_k} \frac{\mathrm{d}x_k}{\mathrm{d}x_j} , \tag{11}$$

which in matrix form translates to

$$G_{ij} = \begin{cases} 1 & i = j \\ \sum_{k=1}^{N} R_{ik} G_{kj} & i \neq j \end{cases}. \tag{12}$$

Hence in our derivation below we first calculate R_{ij} and then, using (12), we seek the terms of G_{ij}.

In the derivation that follows we use the configuration model [4] framework to analyze the underlying network A_{ij}. Under this model, A_{ij} is taken to be a weighted and directed network with arbitrary degree and weight distributions, including also scale-free and other anomalous distributions, but with otherwise random connectivity. Within this framework A_{ij} can capture effects associated with degree/weight distribution, network paths, and individual node (weighted) degrees. The configuration model overlooks, however, several meso-scopic characteristics pertaining to the network's fine-structure, such as degree-degree correlations [5], or clustering, which, in the limit of sparse networks ($\langle k \rangle \ll N \to \infty$) become negligible due to the random connectivity. In Supplementary Note 4 we systematically test the sensitivity of our qualitative and quantitative predictions to the configuration model assumptions, examining the impact of degree-correlations and clustering on the observed flow patterns.

Steady state analysis

To obtain R_{ij} we begin by extracting the fixed point of (1), rewriting (6) as

$$1 - W(x_i) \sum_{k=1}^{N} A_{ik} M_2(x_k) = 0, \tag{13}$$

where

$$W(x_i) = -\frac{M_1(x_i)}{M_0(x_i)}. \tag{14}$$

We further express the sum in (13) as

$$\sum_{k=1}^{N} A_{ik} M_2(x_k) = S_{i,\text{in}} \left(\frac{1}{S_{i,\text{in}}} \sum_{k \in K_{i,\text{in}}} M_2(x_k) \right) = S_{i,\text{in}} \langle M_2(x_k) \rangle_{k \in K_{i,\text{in}}}, \tag{15}$$

where $S_{i,\text{in}} = \sum_{k=1}^{N} A_{ik}$ is node i's incoming weighted degree, and $K_{i,\text{in}} = \{k \in (1, \ldots, N) | A_{ik} \neq 0\}$ is the group of i's nearest (incoming) neighbors. Hence we arrive at

$$W(x_i) = \frac{1}{S_{i,\text{in}} \langle M_2(x_k) \rangle_{k \in K_{i,\text{in}}}}, \tag{16}$$

which, in case $W(x_i)$ is invertible, provides

$$x_i = W^{-1} \left(\frac{1}{S_{i,\text{in}} \langle M_2(x_k) \rangle_{k \in K_{i,\text{in}}}} \right) = W^{-1} \left(\frac{1}{\langle M_2(x_k) \rangle_{k \in K_{i,\text{in}}}} \xi_{i,\text{in}} \right), \tag{17}$$

where $\xi_{i,\text{in}} = 1/S_{i,\text{in}}$ is node i's inverted in-degree. The fact that A_{ij} has little degree-correlations indicates that the nodes in K_i are drawn from a similar distribution as nodes in any other nearest neighbor group K_j. In other words the statistical properties of i's neighborhood are identical to that of any other node's neighborhood. Hence we can simplify the average in (16) to

$$\langle M_2(x_k) \rangle_{k \in K_{i,\text{in}}} = \xi_{i,\text{in}} \sum_{k=1}^{N} A_{ik} M_2(x_k) \approx \frac{1}{N} \sum_{i=1}^{N} \xi_{i,\text{in}} \sum_{k=1}^{N} A_{ik} M_2(x_k) = \langle M_2(x_k) \rangle_{k \in K^{\text{in}}}, \tag{18}$$

where we replaced the average over i's neighbors K_i by an average of all nearest neighbor nodes K. As a result (18) becomes independent of i, allowing us to approximate (16) and (17) as

$$W(x_i) \sim \frac{1}{S_{i,\text{in}}} \tag{19}$$

and hence

$$x_i \sim W^{-1} \left(\frac{1}{S_{i,\text{in}}} \right) = W^{-1} \left(\xi_{i,\text{in}} \right), \tag{20}$$

expressing the steady state x_i in function of i's weighted degree.

Local response R_{ij}

To obtain R_{ij} we induce a small permanent perturbation ∂x_j on j (the source) and follow the asymptotic ($t \to \infty$) response of i (the target), namely we take $x_j \to x_j + \partial x_j$, and use (13) to obtain $x_i \to x_i + \partial x_i$. Note that in R_{ij}, the activities of all other nodes, apart from i and j, remain unchanged (∂ vs. d). Hence following j's perturbation the new steady state to which the system will transition can be obtained from

$$1 + \sum_{\substack{k=1 \\ k \neq j}}^{N} A_{ik} W(x_i + \partial x_i) M_2(x_k) + A_{ij} W(x_i + \partial x_i) M_2(x_j + \partial x_j) = 0, \tag{21}$$

where all but x_i and x_j remain unchanged. We now expand (21) in orders of ∂x_i and ∂x_j, obtaining

$$\begin{aligned} 0 &= 1 + \sum_{k=1}^{N} A_{ik} W(x_i) M_2(x_k) + \sum_{k=1}^{N} A_{ik} W'(x_i) M_2(x_k) \partial x_i \\ &+ A_{ij} W(x_i) M_2'(x_j) \partial x_j + O(\partial x_i \partial x_j), \end{aligned} \tag{22}$$

where

$$W'(x_i) = \left. \frac{\mathrm{d}W}{\mathrm{d}x} \right|_{x = x_i} \tag{23}$$

$$M_2'(x_j) = \left. \frac{\mathrm{d}M_2}{\mathrm{d}x} \right|_{x = x_j}, \tag{24}$$

namely, the derivatives of $W(x_i)$ and $M_2(x_j)$ taken around the system's fixed point. Excluding nonlinear terms in ∂x_i and ∂x_j, and using the steady state condition (13), we bring (22) into the form

$$\left(\sum_{k=1}^{N} A_{ik} W'(x_i) M_2(x_k) \right) \partial x_i + A_{ij} \left(W(x_i) M_2'(x_j) \right) \partial x_j = 0, \tag{25}$$

which, in turn, provides

$$\frac{\partial x_i}{\partial x_j} = -\frac{A_{ij} W(x_i) M_2'(x_j)}{\sum_{k=1}^{N} A_{ik} W'(x_i) M_2(x_k)}. \tag{26}$$

Note, that the pre-factor A_{ij} in the numerator, captures the fact that a non-vanishing local response $R_{ij} \neq 0$ is only observed for directly interacting nodes, namely $R_{ij} \neq 0 \Leftrightarrow A_{ij} \neq 0$. The local response matrix (10) is thus

$$R_{ij} = \left| \frac{\partial x_i / x_i}{\partial x_j / x_j} \right| = \left| \frac{A_{ij} W(x_i) M_2'(x_j) x_j}{x_i \sum_{k=1}^{N} A_{ik} W'(x_i) M_2(x_k)} \right|. \tag{27}$$

To further simplify (27) we follow the same steps leading to (15), expressing the sum in the denominator as

$$\sum_{k=1}^{N} A_{ik} W'(x_i) M_2(x_k) = S_{i,\text{in}} W'(x_i) \langle M_2(x_k) \rangle_{k \in K_{i,\text{in}}}. \tag{28}$$

Using (18) to replace $K_{i,\text{in}}$ with K, independent of i, we arrive at

$$R_{ij} = \left| \frac{A_{ij}}{\langle M_2(x_k) \rangle_{k \in K}} \cdot S_W(x_i) \cdot S_M(x_j), \right| \tag{29}$$

where

$$S_W(x_i) = \frac{W(x_i)}{x_i S_{i,\text{in}} W'(x_i)} \tag{30}$$

$$S_M(x_j) = x_j M_2'(x_j). \tag{31}$$

Equation (29) separates the local response, R_{ij}, into three terms: the prefactor (fraction on left), which is independent of i and j; $S_W(x_i)$ which comprises all terms associated with node i; $S_M(x_j)$, which collects all terms associated with node j. Next, we use this factorization to obtain the scaling of $\mathcal{F}_i$ and $\mathcal{F}_{ij}$ with i and j.

The scaling of $\mathcal{F}_i$

We now use the results obtained above for R_{ij} (29) and G_{ij} (12) to derive the dependence of the flow $\mathcal{F}_i$ on i's weighted degree. Consider a pair of nodes, the source n and the target m, whose response term is given by G_{mn}. To obtain i's contribution to this response we silence i by freezing its activity at its steady-state value x_i, then measuring the i-silenced response term $G_{mn}^{\{i\}}$ (Supplementary Figure. 2b). This allows us to obtain the flow, as defined in the main text

$$\mathcal{F}_{mn}^{\{i\}} = \frac{G_{mn} - G_{mn}^{\{i\}}}{\sum_{m=1}^{N} G_{mn}}, \tag{32}$$

capturing i's contribution to the flow of information between n to m. Summing over m we obtain i's overall contribution to information spread from n

$$\mathcal{F}_{n}^{\{i\}} = \sum_{\substack{m=1 \\ m \neq i}}^{N} \mathcal{F}_{mn}^{\{i\}}. \tag{33}$$

Averaging over n characterizes i's mean contribution to information flow in the network as

$$\mathcal{F}_i = \frac{1}{N-1} \sum_{\substack{n=1 \\ n \neq i}}^{N} \mathcal{F}_{n}^{\{i\}}. \tag{34}$$

Equation (34) defines the contribution of i to the flow of information in the system. Substituting $Z_n = \sum_{m=1}^{N} G_{mn}$ and $Z_n^{\{i\}} = \sum_{m=1}^{N} G_{mn}^{\{i\}}$ it can be written in the form

$$\mathcal{F}_{n}^{\{i\}} = \frac{Z_n - Z_n^{\{i\}}}{Z_n}, \tag{35}$$

as appears in Eq. (4) of the main paper text.

To obtain (34) we first evaluate the silenced term $G_{mn}^{\{i\}}$ in (32). To achieve this we construct it as a composition of two perturbations: Perturbation 1, shifting n's activity by $\mathrm{d}x_n$. This perturbation results in the response $\mathrm{d}x_i$ measured in the activity of node i. Perturbation 2, shifting i's activity by $-\mathrm{d}x_i$, in effect, canceling the impact of perturbation 1 on i. Hence Perturbation 1 represents the

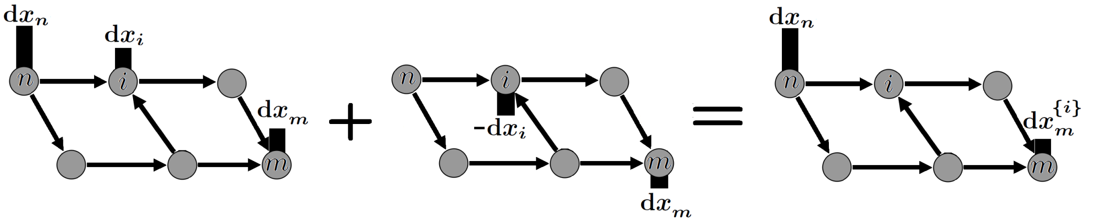

Supplementary Figure 1: **Superposition of perturbations**. We construct $G_{mn}^{\{i\}}$ from two perturbations (signals): the real signal $\mathrm{d}x_n$, which generates the response $\mathrm{d}x_m$ joint with a hypothetical signal $-\mathrm{d}x_i$, which, in effect freezes i's activity, annihilating its response to the n-signal. The superposition of the real and hypothetical signals provides the i-frozen response $\mathrm{d}x_m^{\{i\}}$.

actual perturbation induced on the source node n, and Perturbation 2 represents a hypothetical perturbation, which effectively silences i's response (Supplementary Figure. 1). In the linear regime (small perturbations) the sum of both perturbations provides $G_{mn}^{\{i\}}$. Consider the response $\mathrm{d}x_m^{\{i\}}$, representing m's response to the composition of Perturbations 1 and 2. We can write it as

$$\mathrm{d}x_m^{\{i\}} = \mathrm{d}x_m - \frac{\mathrm{d}x_m}{\mathrm{d}x_i}\mathrm{d}x_i, \tag{36}$$

where $\mathrm{d}x_m$ is m's response to Perturbation 1 (without silencing i) and the second term subtracts i's contribution through the hypothetical Perturbation 2. Dividing both sides of (36) by $\mathrm{d}x_n$ we write

$$\frac{\mathrm{d}x_m^{\{i\}}}{\mathrm{d}x_n} = \frac{\mathrm{d}x_m}{\mathrm{d}x_n} - \frac{\mathrm{d}x_m}{\mathrm{d}x_i}\cdot\frac{\mathrm{d}x_i}{\mathrm{d}x_n}, \tag{37}$$

which, by appropriately dividing both the numerators and the denominators by x_m, x_n and x_i, becomes

$$G_{mn}^{\{i\}} = G_{mn} - G_{mi}G_{in}. \tag{38}$$

Substituting (38) into $\mathcal{F}_n^{\{i\}}$ (33) we write

$$\mathcal{F}_n^{\{i\}} = \sum_{\substack{m=1\\m\neq i}}^{N} \frac{G_{mi}G_{in}}{\sum_{m=1}^{N} G_{mn}} = G_{in}\sum_{\substack{m=1\\m\neq i}}^{N} \frac{G_{mi}}{\sum_{m=1}^{N} G_{mn}}. \tag{39}$$

allowing us to express the flow in terms of the response matrix G. Consider the response G_{mi} on the r.h.s. of Eq. (39), capturing i's impact on m's activity. We can approximate this response using an effective chain rule

$$G_{mi} \approx \sum_{k \in K_{i,\text{out}}} R_{ki} G_{mk}, \tag{40}$$

describing first i's impact on its nearest neighbors R_{ki} ($k \in K_{i,\text{out}}$), and then the impact of these neighbors on m, G_{mk}. Next, we decompose the sum in (40) as

$$G_{mi} \approx \frac{1}{|k_{i,\text{out}}|} \sum_{k \in K_{i,\text{out}}} R_{ki} \sum_{k \in K_{i,\text{out}}} G_{mk} = \langle G_{mk} \rangle_{k \in K_{i,\text{out}}} \sum_{k \in K_{i,\text{out}}} R_{ki}, \tag{41}$$

exact in the limit where the terms in the summation are uncorrelated. Indeed, for a pair of arbitrary nodes i and m, the response terms R_{ki}, a neighbor's k response to i, and G_{mk}, a random node's m response to k, are assumed to exhibit little correlation, and hence $\langle R_{ki} G_{mk} \rangle \approx \langle R_{ki} \rangle \langle G_{mk} \rangle$, allowing us to reverse the order sum/product. We further use the randomness of A_{ij}, in which there is little degree correlations, to substitute $\langle G_{mk} \rangle_{k \in K_{i,\text{out}}} \approx \langle G_{mk} \rangle_{k \in K}$, namely that the average response of all nodes to i's nearest neighbors $K_{i,\text{out}}$ can be replaced by that of all nearest neighbors, K (see Eq. (18)). Using G_{mi} (41) in (39) provides us with

$$\mathcal{F}_n^{\{i\}} = \left(\sum_{\substack{m=1 \\ m \neq i}}^{N} \frac{\langle G_{mk} \rangle_{k \in K^{\text{out}}}}{\sum_{m=1}^{N} G_{mn}} \right) G_{in} \cdot \sum_{k \in K_{i,\text{out}}} R_{ki}, \tag{42}$$

leading to

$$\mathcal{F}_n^{\{i\}} \sim G_{in} \cdot \sum_{k \in K_{i,\text{out}}} R_{ki}, \tag{43}$$

where in the final step we omitted all terms that are independent of i. Next we express G_{in} in (43) using (12), providing us with

$$\mathcal{F}_n^{\{i\}} \sim \sum_{l \in K_{i,\text{in}}} R_{il} G_{ln} \cdot \sum_{k \in K_{i,\text{out}}} R_{ki} \sim \sum_{l \in K_{i,\text{in}}} R_{il} \cdot \sum_{k \in K_{i,\text{out}}} R_{ki}, \tag{44}$$

where once again we factored out terms independent of i. Note that the term $\sum_{l\in K_{i,\text{in}}} R_{il}$ sums over all $k_{i,\text{in}} = |K_{i,\text{in}}|$ incoming perturbations to node i. Similarly $\sum_{k\in K_{i,\text{out}}} R_{ki}$ includes all $k_{i,\text{out}} = |K_{i,\text{out}}|$ perturbations outgoing from i to its nearest neighbors. Hence we write

$$\mathcal{F}_n^{\{i\}} \sim k_{i,\text{in}} k_{i,\text{out}} \langle R_{il}\rangle_{l\in K_{i,\text{in}}} \langle R_{ki}\rangle_{k\in K_{i,\text{out}}}. \tag{45}$$

Next, averaging over n, and taking R_{ij} from (29), we obtain $\mathcal{F}_i$ (34), which takes the form

$$\begin{aligned}\mathcal{F}_i \quad &\sim \quad k_{i,\text{out}} k_{i,\text{in}} \left\langle \frac{A_{il}}{\langle M_2(x_n)\rangle_{n\in K}} S_W(x_i) S_M(x_l) \right\rangle_{l\in K_{i,\text{in}}} \left\langle \frac{A_{ki}}{\langle M_2(x_n)\rangle_{n\in K}} S_W(x_k) S_M(x_i) \right\rangle_{k\in K_{i,\text{out}}} \\ &= \left\langle \frac{A_{il}}{\langle M_2(x_n)\rangle_{n\in K}} S_M(x_l) \right\rangle_{l\in K_{i,\text{in}}} \left\langle \frac{A_{ki}}{\langle M_2(x_n)\rangle_{n\in K}} S_W(x_k) \right\rangle_{k\in K_{i,\text{out}}} \\ &\cdot \quad k_{i,\text{out}} k_{i,\text{in}} S_W(x_i) S_M(x_i).\end{aligned} \tag{46}$$

As before, we only keep the terms that depend on i and therefore contribute to the scaling of $\mathcal{F}_i$ with k_i, providing us with

$$\mathcal{F}_i \sim k_{i,\text{out}} k_{i,\text{in}} S_W(x_i) S_M(x_i). \tag{47}$$

Next we take $S_W(x_i)$ and $S_M(x_i)$ from (30) and (31), finding

$$\mathcal{F}_i \sim k_{i,\text{out}} k_{i,\text{in}} x_i M_2'(x_i) \frac{W(x_i)}{S_{i,\text{in}} x_i W'(x_i)} = k_{i,\text{in}} k_{i,\text{out}} \frac{1}{S_{i,\text{in}}} M_2'(x_i) \frac{W(x_i)}{W'(x_i)}. \tag{48}$$

At the steady state, Eq. (19) predicts that $W(x_i) \sim 1/S_{i,\text{in}}$, and hence Eq. (48) provides us with

$$\mathcal{F}_i \sim k_{i,\text{out}} k_{i,\text{in}} \left(\frac{1}{S_{i,\text{in}}}\right)^2 \frac{M_2'(x_i)}{W'(x_i)}. \tag{49}$$

We now use (19) to write

$$W'(x_i) \sim \frac{\partial\left(\frac{1}{S_{i,\text{in}}}\right)}{\partial x_i} = \frac{\partial \xi_{i,\text{in}}}{\partial x_i} = \frac{\partial \xi_{i,\text{in}}}{\partial W^{-1}(\xi_{i,\text{in}})}\,, \tag{50}$$

and (20) to write

$$M_2'(x_i) \sim \frac{\partial M_2\big(W^{-1}(\xi_{i,\text{in}})\big)}{\partial \xi_{i,\text{in}}} \cdot \frac{\partial \xi_{i,\text{in}}}{\partial W^{-1}(\xi_{i,\text{in}})}\,. \tag{51}$$

Taken together (49) becomes

$$\begin{aligned}\mathcal{F}_i &\sim k_{i,\text{out}} k_{i,\text{in}} \left(\frac{1}{S_{i,\text{in}}}\right)^2 \left(\frac{\partial \xi_{i,\text{in}}}{\partial W^{-1}(\xi_{i,\text{in}})}\right)^{-1} \frac{\partial M_2\big(W^{-1}(\xi_{i,\text{in}})\big)}{\partial \xi_{i,\text{in}}} \frac{\partial \xi_{i,\text{in}}}{\partial W^{-1}(\xi_{i,\text{in}})} \\ &= k_{i,\text{out}} k_{i,\text{in}} \left(\frac{1}{S_{i,\text{in}}}\right)^2 \frac{\partial M_2\big(W^{-1}(\xi_{i,\text{in}})\big)}{\partial \xi_{i,\text{in}}}. \end{aligned} \tag{52}$$

This result indicates that $\mathcal{F}_i$ depends on node i's properties through both its unweighted degrees $k_{i,\text{in}}$ and $k_{i,\text{out}}$ and its weighted degree $S_{i,\text{in}}$. However, to obtain the specific function for $\mathcal{F}_i$, we must also consider the contribution of the derivative term on the r.h.s. of (52), which involves the node's weighted degree through $\xi_{i,\text{in}} = 1/S_{i,\text{in}}$. This term, involving the functions $W(x)$ and $M_2(x)$, captures the role of the dynamics in determining $\mathcal{F}_i$. Note that the argument of the function is $\xi_{i,\text{in}}$, which in the limit of large $S_{i,\text{in}}$ approaches zero. Under these conditions we can take only the leading terms in its expansion around zero. Hence, using the Hahn series expansions (4) we express the function $M_2(W^{-1}(x))$ as a power series in the form

$$M_2\big(W^{-1}(x)\big) = \sum_{n=0}^{\infty} C_n (x - x_0)^{\Gamma(n)}, \tag{53}$$

where, as before, the powers $\Gamma(n)$ represent a countable well-ordered set in ascending order with n. In the limit of large $S_{i,\text{in}}$, we have $\xi_{i,\text{in}} = 1/S_{i,\text{in}} \to 0$, allowing us to expand the derivative of $M_2(W^{-1}(x))$ in (52) up to leading order terms in

ξ_i. The result is

$$\left.\frac{\partial M_2(W^{-1}(\xi_{i,\text{in}}))}{\partial \xi_{i,\text{in}}}\right|_{\xi_{i,\text{in}}\to 0} \sim \begin{cases} {\xi_{i,\text{in}}}^{\Gamma(0)-1} & \Gamma(0) \neq 0 \\ {\xi_{i,\text{in}}}^{\Gamma(1)-1} & \Gamma(0) = 0 \end{cases} . \tag{54}$$

Substituting (54) into (52), and replacing $\xi_{i,\text{in}}$ with $1/S_{i,\text{in}}$ we obtain

$$\mathcal{F}_i \sim k_{i,\text{out}} k_{i,\text{in}} \left(S_{i,\text{in}}\right)^{\omega-2} \tag{55}$$

where

$$\omega = \begin{cases} 1-\Gamma(0) & \Gamma(0) \neq 0 \\ 1-\Gamma(1) & \Gamma(0) = 0 \end{cases} . \tag{56}$$

In case the weights A_{ij} are randomly distributed, independent of $k_{i,\text{out}}$ or $k_{i,\text{in}}$, we have $S_{i,\text{out}} \sim k_{i,\text{out}}$ and $S_{i,\text{in}} \sim k_{i,\text{in}}$. Indeed, under these conditions the weighted degree, *e.g.*, $S_{i,\text{out}}$, constitutes a sum of $k_{i,\text{out}}$ independent random variables, and hence, on average scales linearly with $k_{i,\text{out}}$. Focusing only on the scaling of $\mathcal{F}_i$, we can thus replace the unweighted degrees with the appropriate weighted degrees, obtaining

$$\boxed{\mathcal{F}_i \sim S_{i,\text{out}} S_{i,\text{in}}^{\omega-1},} \tag{57}$$

arriving at Eq. (6) of the main, validated in Fig. 2y.

The results (55) and (57) expose the non-trivial relationship between the dynamic flow $\mathcal{F}_i$ and the network structure A_{ij}. Indeed, Eq. (55) receives as input the topological characteristics of all nodes as expressed by their degrees $k_{i,\text{in}}$, $k_{i,\text{out}}$ and $S_{i,\text{in}}$, and provides as output their dynamic contribution to the flow of information $\mathcal{F}_i$. However, the translation of the node's degree into information flow is dynamics-dependent, as the exponent ω in (55) is determined by the system's dynamics $\mathbf{M}$ in Eq. (1). Hence, even if two systems share the same topology, their actual patterns of information flow may significantly differ, depending on the leading terms of (53).

Most importantly, the exponent ω is analytically derived directly from the system's dynamics, specifically from the functional form of $M_0(x)$, $M_1(x)$ and $M_2(x)$:

first we extract $W(x)$ as $W(x) = -M_1(x)/M_0(x)$; then, by inversion, we obtain $W^{-1}(x)$; finally we substitute the inverted function into $M_2(x)$ to obtain $M_2(W^{-1}(x))$ as appears in (53). Taking the two leading powers in this expansion, $\Gamma(0)$ and $\Gamma(1)$, provides us with ω through (56). Hence ω can be analytically extracted directly from the system's dynamic model. Also note, that ω is independent of the coefficients C_n in (53), and hence it is insensitive to the specific model parameters, just to its intrinsic functional form, as expressed through the powers $\Gamma(n)$.

Next, we consider the special case of an undirected network, where $k_{i,\text{in}} = k_{i,\text{out}} = k_i$ for all nodes. Note that the weighted degrees may still differ $S_{i,\text{in}} \neq S_{i,\text{out}}$, since the weight A_{ij} may be different from A_{ji}. If, however, the weights are distributed at random, such that $P(A_{ij} = a | k_i, k_j) = P(A_{ij} = a)$, namely that the weights between nodes are independent of their degrees, we have $S_{i,\text{in}} \sim S_{i,\text{out}} \sim k_i$, since $S_{i,\text{in}}$ and $S_{i,\text{out}}$ both represent a sum of k_i independent random weights. This allows us, for simplicity, to use the undirected weighted degree S_i, which is proportional (on average) to both $S_{i,\text{in}}$ and $S_{i,\text{out}}$. Under these circumstances we have $k_{i,\text{in}} k_{i,\text{out}} \sim S_i^2$, which in (54) provides

$$\boxed{\mathcal{F}_i \sim S_i^{\omega},} \tag{58}$$

as appears in Eq. (10) of the main text and validated in Fig. 3. Equation (58) expresses the rules that govern the flow of information in the network through a single exponent ω:

(i) This exponent links between a node's degree and its dynamic contribution to the flow. If ω is large, the hubs control the main channels of information flow in the network; if $\omega = 0$ the contribution to the flow is independent of degree, with hubs and small nodes equally contributing to the dynamic flow; finally, in case $\omega < 0$ the hubs contribute less than the low degree nodes, as information flow is directed mainly by the peripheral nodes, bypassing the well connected hubs. Hence, by predicting ω we gain crucial insight into the patterns of dynamic flow in a complex network.

(ii) The fact that ω can be extracted directly from the system's dynamics shows

that the patterns of information flow are independent of the network topology. Indeed, the flow through a specific node clearly depends on A_{ij} through S_i, however the relationship between S_i (topology) and $\mathcal{F}_i$ (dynamic behavior), which is captured by ω, is a characteristic of the dynamic model, as encapsulated in (53). Hence ω is intrinsic to the system's dynamics $M_0(x)$, $M_1(x)$ and $M_2(x)$.

(iii) The specific value of ω is determined only by the two leading powers of (53), independent of the specific coefficients (C_n) or of higher order terms. This captures a universality across diverse forms of dynamics. Indeed, even dynamic models from highly distinct domains may exhibit a similar flow pattern, if the leading powers in the expansion of $M_2(W^{-1}(x))$ happen to coincide.

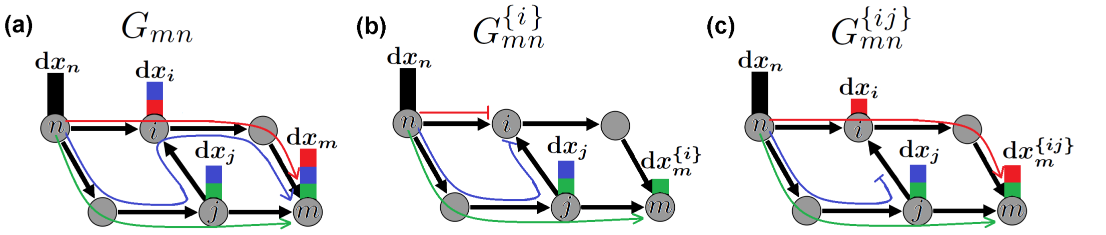

Supplementary Figure 2: **Freezing a node or blocking an edge**. (a) A signal $\mathrm{d}x_n$ propagates through all paths to impact x_m. The response $\mathrm{d}x_m$ is a result of the contribution of all paths(red, green, blue) linking m to n. (b) Freezing node i terminates the flow of information through the red and blue paths, preserving only the green contribution to $\mathrm{d}x_m$. The reduction in $\mathrm{d}x_m$ captures i's contribution to the flow from n to m. (c) Blocking A_{ij} does not freeze the i, j nodes, but only blocks the part of i's response flowing via the direct link from j. The red and green pathways remain active, but the blue path is now blocked. The resulting reduction in $\mathrm{d}x_m$, namely the blue contribution quantifies the information flow through A_{ij}.

The scaling of $\mathcal{F}_{ij}$

Consider the dynamic equation (1). For each node i, the equation accounts for the incoming information from all of its $k_{i,\text{in}}$ incoming neighbors, as expressed through

the interaction term $\sum_{j=1}^{N} A_{ij} M_1\big(x_i(t)\big) M_2\big(x_j(t)\big)$. Hence the coupling term A_{ij} represents an incoming (weighted) link from j to i, namely $A_{ij} \equiv A_{i \leftarrow j}$. To obtain the contribution of this edge to the flow from the source n towards the target m, we must restrict A_{ij} from propagating information from j to i. The signal $\mathrm{d}x_n$ will then spread through the remaining paths of the network, but will not be propagated by the link A_{ij} (Supplementary Figure. 2c). Such blocking of spread through a single link can be achieved by exclusively freezing the activity $x_j(t)$ in the ith equation. Indeed, this will result in i "seeing" the unperturbed steady state value of x_j, effectively receiving no information on the signal $\mathrm{d}x_n$ through the incoming link from j. To be explicit, we write Eq. (1) with a perturbed source node $x_n(t) = x_n + \mathrm{d}x_n$, and a blocked A_{ij}, by changing the ith equation to

$$\frac{\mathrm{d}x_i}{\mathrm{d}t} = M_0\big(x_i(t)\big) + \sum_{\substack{q=1 \\ q \neq j}}^{N} A_{iq} M_1\big(x_i(t)\big) M_2\big(x_q(t)\big) + A_{ij} M_1\big(x_i(t)\big) M_2(x_j), \tag{59}$$

in which the relevant interaction term $A_{ij} M_1(x_i(t)) M_2(x_j)$ expresses the unperturbed steady state of j. Note, that in this process: (i) we do not remove the A_{ij} edge, as that would result in altering the topology and consequently changing the states x_i of all nodes; (ii) we do not freeze the activity of nor j neither i. Node j's equation is unchanged, hence it still responds to the signal $\mathrm{d}x_n$; node i may still receive information on the spreading signal from n through other paths not involving A_{ij}. Hence (59) ensures that only the part of the spread that is propagated via A_{ij} is blocked, preserving the contribution of all other paths to the spread. In simple terms, we only block x_i from being impacted by $\mathrm{d}x_j$, the role played by A_{ij} in spreading the signal $\mathrm{d}x_n$. Solving (1) and substituting the ith equation with the blocked (59) provides us with $G_{mn}^{\{ij\}}$, representing the response of all target (m) nodes to the source's (n) signal, excluding the contribution of A_{ij}. Following our previous definition this allows us to evaluate the flow from m to n through A_{ij} as

$$\mathcal{F}_{mn}^{\{ij\}} = \frac{G_{mn} - G_{mn}^{\{ij\}}}{\sum_{m=1}^{N} G_{mn}}, \tag{60}$$

similar to $\mathcal{F}_n^{\{i\}}$ in (33). To obtain the global contribution of the link $A_{ij}$to the

spread we average over all pairs n, m arriving at

$$\mathcal{F}_{ij} = \frac{1}{N}\sum_{n=1}^{N}\sum_{m=1}^{N}\mathcal{F}_{mn}^{\{ij\}}. \tag{61}$$

To derive the analytical expression for $\mathcal{F}_{ij}$ let us first understand the mechanism by which blocking A_{ij} affects G_{mn}. To impact m's activity, the signal $\mathrm{d}x_n$ must pass through all pathways leading from n to m, specifically ones crossing A_{ij}. In this process, node j responds to the signal as $x_j \to x_j + \mathrm{d}x_j$, causing node i to respond as $x_i \to x_i + \mathrm{d}x_i$, eventually reaching the target m. Blocking A_{ij} reduces (but not eliminates) the magnitude of i's response, resulting in a diminished response $\mathrm{d}x_m$ of all remaining nodes. The reduction of i's response can be evaluated by

$$\mathrm{d}x_i - \mathrm{d}x_i^{\{ij\}} = \frac{\partial x_i}{\partial x_j}\mathrm{d}x_j, \tag{62}$$

where the partial derivative (∂) captures the fact that only the direct information from j to i is blocked, but indirect paths remain active. Indeed, blocking the i, j link does not prohibit information transfer from j to i through indirect pathways. Therefore, we can express the response of the target node m as a superposition of two perturbations, the actual perturbation $\mathrm{d}x_n$ and a hypothetical perturbation $-\frac{\partial x_i}{\partial x_j}\mathrm{d}x_j$, to captures the reduction in $\mathrm{d}x_i$ as a result of blocking A_{ij}. This results in an analogous Eq. to (36), providing

$$\mathrm{d}x_m^{\{ij\}} = \mathrm{d}x_m - \frac{\mathrm{d}x_m}{\mathrm{d}x_i}\frac{\partial x_i}{\partial x_j}\mathrm{d}x_j. \tag{63}$$

Equation (63) should be read as follows:

$$\begin{pmatrix}\text{The response}\\ \text{of } m \text{ under}\\ \text{blocking } A_{ij}\end{pmatrix} = \begin{pmatrix}\text{The full re-}\\ \text{sponse of } m\\ \text{to } \mathrm{d}x_n\end{pmatrix} - \begin{pmatrix}m\text{'s response}\\ \text{to changes in}\\ i\end{pmatrix}\begin{pmatrix}\text{The change}\\ \text{in } \mathrm{d}x_i \text{ due to}\\ \text{blocking } A_{ij}\end{pmatrix}, \tag{64}$$

where the last term is taken form (62). Next we express (63) in matrix form as

$$G_{mn}^{\{ij\}} = G_{mn} - G_{mi}R_{ij}G_{jn}, \tag{65}$$

which in (61) provides

$$\mathcal{F}_{ij} = \frac{1}{N} \left(\sum_{n=1}^{N} \sum_{m=1}^{N} \frac{G_{mi} G_{jn}}{\sum_{m=1}^{N} G_{mn}} \right) R_{ij}, \tag{66}$$

where we have taken $\mathcal{F}_{mn}^{\{ij\}}$ from (60). As we are interested in the scaling relationship between $\mathcal{F}_{ij}$ and the node characteristics of i and j, namely their weighted/unweighted in/out degrees, we can omit from our derivation all terms that are independent of i and j, *e.g.*, the summation over G_{mn} in the denominator of (66). Hence our goal reduces to obtaining the i dependence of $\sum_m G_{mi}$, the j dependence of $\sum_n G_{jn}$ and the i, j dependence of R_{ij}.

First, let us discuss the scaling behavior of $\sum_m G_{mi}$, which captures the total response of all nodes $m = 1, \ldots, N$ to i's perturbation. This response can be evaluated in two steps: first measuring $\sum_m R_{mi}$, namely the direct response of i's nearest neighbors, then multiplying that by the response of all remaining nodes to i's perturbed neighbors. However, as A_{ij} features little degree correlations, the latter is independent of i. Indeed, the propagation from i's nearest neighbors to the rest of the network, depends on the properties of i's nearest neighbors, then on the properties of its next nearest neighbors and so on. For a random A_{ij} these are all independent of i[4]. Therefore we can write

$$\sum_{m=1}^{N} G_{mi} = c \sum_{m=1}^{N} R_{mi}, \tag{67}$$

where the constant c captures the propagation from i's direct neighbors to the rest of the network. As c is independent of i, we omit it, writing

$$\sum_{m=1}^{N} G_{mi} \sim \sum_{m=1}^{N} R_{mi}, \tag{68}$$

A similar argument provides us with

$$\sum_{n=1}^{N} G_{jn} \sim \sum_{m=1}^{N} R_{jn}. \tag{69}$$

Note that R_{ij} vanishes if i is not directly linked to j, and hence the summations in (68) and (69) include only nearest outgoing neighbors of i or nearest incoming neighbors of j, respectively. We can express these two equations using the average nearest neighbor responses as

$$\begin{cases} \sum_{m=1}^{N} R_{mi} = k_{i,\mathrm{out}} \langle R_{mi} \rangle_{m \in K_{i,\mathrm{out}}} \\ \sum_{n=1}^{N} R_{jn} = k_{j,\mathrm{in}} \langle R_{jn} \rangle_{n \in K_{j,\mathrm{in}}} \end{cases} . \tag{70}$$

Gathering all the terms that contribute to the i, j dependence of $\mathcal{F}_{ij}$, we simplify (66) to

$$\mathcal{F}_{ij} \sim k_{i,\mathrm{out}} k_{j,\mathrm{in}} R_{ij} \langle R_{mi} \rangle_{m \in K_{i,\mathrm{out}}} \langle R_{jn} \rangle_{n \in K_{j,\mathrm{in}}} . \tag{71}$$

Referring back to Eq. (29) we write

$$R_{ij} = \left| \frac{A_{ij}}{\langle M_2(x_n) \rangle} S_W(x_i) S_M(x_j) \right| , \tag{72}$$

recalling that

$$S_W(x_i) = \frac{W(x_i)}{x_i S_{i,\mathrm{in}} W'(x_i)} \tag{73}$$

$$S_M(x_j) = x_j M_2'(x_j). \tag{74}$$

This allows us to express (71) as

$$\mathcal{F}_{ij} \sim A_{ij} k_{i,\mathrm{out}} k_{j,\mathrm{in}} S_W(x_i) S_M(x_j) \langle A_{mi} S_W(x_m) \rangle_{m \in K_{i,\mathrm{out}}} \cdot S_M(x_i) S_W(x_j) \langle A_{jn} S_M(x_n) \rangle_{n \in K_{j,\mathrm{in}}} , \tag{75}$$

where, as before, we only keep terms that are explicitly dependent on i or j. Equation (75) includes two averages of the form $\langle \cdot \rangle$ in which we average over i or j's nearest incoming/outgoing neighbors. While, such averages, carried over

the neighborhoods of i or j, may, in general, depend on i or on j, here, under the absence of degree correlations, we assume that the statistical properties of a node's neighborhood are independent of the node itself. Therefore the $\langle \cdot \rangle$ terms in (75) do not contribute to the i,j scaling, allowing us to further simplify $\mathcal{F}_{ij}$ to

$$\mathcal{F}_{ij} \sim A_{ij} k_{i,\text{out}} k_{j,\text{in}} S_W(x_i) S_M(x_j) S_M(x_i) S_W(x_j). \tag{76}$$

Using (47) we substitute $S_W(x_i)S_M(x_i)$ with $\mathcal{F}_i / k_{i,\text{out}} k_{i,\text{in}}$ and take $\mathcal{F}_i \sim k_{i,\text{out}} k_{i,\text{in}} S_{i,\text{in}}^{\omega-2}$ as in Eq. (55). Doing the same for j, namely $\mathcal{F}_j \sim k_{j,\text{out}} k_{j,\text{in}} S_{j,\text{in}}^{\omega-2}$ we bring (76) to the form

$$\mathcal{F}_{ij} \sim A_{ij} k_{i,\text{out}} k_{j,\text{in}} S_{i,\text{in}}^{\omega-2} S_{j,\text{in}}^{\omega-2}, \tag{77}$$

which can be written as

$$\mathcal{F}_{ij} \sim A_{ij} k_{i,\text{out}} k_{j,\text{in}} S_{i,\text{in}}^{\xi-1} S_{j,\text{in}}^{\xi-1}, \tag{78}$$

where

$$\xi = \omega - 1. \tag{79}$$

Finally, if weights are randomly distributed, a node's weighted degree scales linearly with its number of neighbors, hence $S_{i,\text{out}} \sim k_{i,\text{out}}$ and $S_{j,\text{in}} \sim k_{j,\text{in}}$, bringing $\mathcal{F}_{ij}$ to its final form

$$\boxed{\mathcal{F}_{ij} \sim A_{ij} S_{i,\text{out}} S_{i,\text{in}}^{\xi-1} S_{j,\text{in}}^{\xi},} \tag{80}$$

as appears in the main text in Eq. (9) and validated in Fig. 2z.

Obtaining ω, ξ and the universality class - step by step.

- Separate your dynamics into $\mathbf{M} = (M_0(x), M_1(x), M_2(x))$.
- Write $W(x) = -M_1(x)/M_0(x)$ and obtain its inverse $W^{-1}(x)$.
- Expand the composite function $M_2(W^{-1}(x))$ as a power series around $x = 0$ and extract its two leading powers $\Gamma(0)$ and $\Gamma(1)$.

- Obtain w from (56); $\xi = \omega - 1$.
- $\omega > 0 \rightarrow$ Degree driven; $\omega = 0 \rightarrow$ Homogeneous; $\omega < 0 \rightarrow$ Degree averting.

Permanent vs. transient perturbations

Perturbative analyses represent a fundamental parading to track the propagation of information among interacting particles. A classic example, which is closely related to our approach, is linear stability analysis, where the stability of a nonlinear system is evaluated by observing its response to a local perturbation $\mathrm{d}x_n(t)$, which can either relax to the original fixed-point (stability), diverge (instability) or express cyclic/chaotic behavior. The main difference is, however, that our linear response matrix G_{mn} is obtained by setting a permanent perturbation, which does not relax in time $\mathrm{d}x_n$. Under these conditions, even if the system is at a stable fixed-point, the permanent perturbation will force it into a new perturbed state, in which all nodes have drifted away to some extent from their original state. In mathematical terms, the linear stability framework introduces a perturbed initial condition ($x_n(t = 0) = x_n + \mathrm{d}x_n$), while our perturbations constitute a perturbed boundary condition ($x_n(t) = x_n + \mathrm{d}x_n$, for all t).

Two main motivations underlie our focus on permanent perturbations:

Empirical relevance. Most relevant systems reside in the vicinity of a permanent fixed-point, and are hence, in the long term, insensitive to instantaneous perturbations. Such perturbations decay in time and very rarely penetrate deep into the network. Under these conditions, the amount of information traversing through a selected node, depends more on the system's stability than on that node's dynamic characteristics. For instance, if the perturbation signal decays rapidly, a distant node would barely even be exposed to the propagating information. Permanent perturbations, on the other hand, behave as a constant source of propagating information, allowing us to test the role of all network paths in propagating the information flow.

Practical observations. Permanent perturbations represent a common procedure for controlled observation of many real complex systems. For instance, in sub-cellular biology, genetic knockouts a permanent perturbation - allow us to

observe information flow in gene regulation. Similarly, in social systems stubborn agents disseminate information by sticking to their unchanged opinions. Permanent perturbations are also relevant in naturally occurring settings, such as component failure in technological networks, or species extinction in ecological networks all time invariant perturbations that force the system to respond. Hence, from an empirical observation perspective, permanent perturbations represent a highly relevant premise for information flow analysis.

Supplementary Note 2

Analysis of dynamic models

We tested our predictions of ω and ξ on several commonly used dynamic models. Below we show in detail the specific analysis of each of the models we used.

Epidemic spread $\mathbb{E}$

To describe the spread of infectious diseases we used the susceptible-infected-susceptible (SIS) model [6–8], in which each node exhibits two distinct states: susceptible (S), and infected (I). A susceptible node may become infected through interaction with an infected individual at a rate R

$$I + S \xrightarrow{R} 2I. \tag{81}$$

An infected node recovers at a rate B, becoming susceptible again

$$I \xrightarrow{B} S. \tag{82}$$

Denoting the probability of infection of node i by $0 \le x_i(t) \le 1$, the SIS model maps into supplementary equation (1) with

$$\begin{aligned} M_0\big(x_i(t)\big) &= -Bx_i(t) \\ M_1\big(x_i(t)\big) &= R(1 - x_i(t)) \\ M_2\big(x_j(t)\big) &= x_j(t) \end{aligned} \ , \tag{83}$$

together providing

$$\frac{dx_i(t)}{dt} = -Bx_i(t) + \sum_{j=1}^{N} A_{ij} R\big(1 - x_i(t)\big)x_j(t). \tag{84}$$

The first term on the r.h.s. describes the process of recovery, which occurs at a rate proportional to B and to the probability of being infected $x_i(t)$; the second term describes the process of infection, which depends on i's neighbor j being infected (probability $x_j(t)$) and on i being susceptible (probability $1 - x_i(t)$).

In the SIS model we have (14)

$$W(x) = \frac{R(1-x)}{Bx}, \tag{85}$$

whose inverse is given by

$$W^{-1}(x) = \frac{R}{R - Bx}. \tag{86}$$

Together with $M_2(x) = x$ (83), we obtain the composite function (53)

$$M_2\big(W^{-1}(x)\big) = \frac{R}{R - Bx}, \tag{87}$$

which can be expanded into a Hahn series as

$$M_2\big(W^{-1}(x)\big)\Big|_{x \to 0} \sim 1 + \frac{B}{R}x + O(x^2). \tag{88}$$

In this expansion we have a vanishing leading power $\Gamma(0) = 0$ and the next leading power $\Gamma(1) = 1$. Hence, using (56) we predict that

$$\omega = 1 - \Gamma(1) = 0 \tag{89}$$

and (79)

$$\xi = \omega - 1 = -1. \tag{90}$$

Both predictions are found to be in perfect agreement with results presented in the main paper on a set of diverse real and model networks (Figs. 2 and 3 in main text).

Biochemical dynamics $\mathbb{B}$

Biochemical processes within living cells are mediated by protein-protein interactions (PPI) [9–11], in which proteins bind to form protein complexes. Here we consider a PPI network, with the following biochemical reactions

$$\begin{aligned}
&\varnothing \xrightarrow{F_i} X_i \\
&X_i \xrightarrow{B_i} \varnothing \\
&X_i + X_j \underset{U_{ij}}{\overset{R_{ij}}{\rightleftharpoons}} X_i X_j \\
&X_i X_j \xrightarrow{Q_{ij}} \varnothing .
\end{aligned} \tag{91}$$

The first reaction describes the influx of proteins synthesized by the DNA at a rate F_i; the second reaction describes protein degradation at rate B_i; the third reaction captures the second order interaction between selected pairs of proteins i and j, forming the hetero-dimer $X_i X_j$. The binding/unbinding, rates are given by R_{ij} and U_{ij} respectively, which vanish in case the pair i and j does not interact, together outlining the sparse PPI network; finally, the hetero-dimer $X_i X_j$ undergoes degradation at a rate Q_{ij}. We use mass-action-kinetics [9, 12] to model (91) via (1) as

$$\begin{cases} \dfrac{\mathrm{d}x_i(t)}{\mathrm{d}t} = F_i - B_i x_i(t) + \sum_{j=1}^{N} U_{ij} x_{ij}(t) - \sum_{j=1}^{N} R_{ij} x_i(t) x_j(t) \\ \dfrac{\mathrm{d}x_{ij}(t)}{\mathrm{d}t} = R_{ij} x_i(t) x_j(t) - (U_{ij} + Q_{ij}) x_{ij}(t), \end{cases} \tag{92}$$

where $x_i(t)$ represents the concentration of protein X_i at time t and $x_{ij}(t)$ represents the concentration of the hetero-dimer $X_i X_j$. Next we assume that the hetero-dimer responds quickly to changes in the concentrations of its monomer proteins, being at quazy-steady state, expressed in (92) through $\dfrac{\mathrm{d}x_{ij}(t)}{\mathrm{d}t} = 0$. We obtain

$$x_{ij}(t) = \frac{R_{ij}}{U_{ij} + Q_{ij}} x_i(t) x_j(t), \tag{93}$$

which in (92) provides

$$\frac{dx_i(t)}{dt} = F_i - B_i x_i(t) - \sum_{j=1}^{N} A_{ij} x_i(t) x_j(t), \tag{94}$$

where

$$A_{ij} = \frac{U_{ij} R_{ij}}{U_{ij} + Q_{ij}} - R_{ij} = \frac{Q_{ij} R_{ij}}{U_{ij} + Q_{ij}} \tag{95}$$

is the effective rate constant for the interaction of X_i and X_j. supplementary equation (94) expresses sub-cellular biochemical dynamics within the form of (1) with

$$\begin{aligned} M_0(x_i(t)) &= F - Bx_i(t) \\ M_1(x_i(t)) &= -x_i(t) \\ M_2(x_j(t)) &= x_j(t), \end{aligned} \tag{96}$$

where the weighted A_{ij} network is composed of the system's rate constants via (95). For simplicity we take $F = \langle F_i \rangle$ and $B = \langle B_i \rangle$, the average influx and degradation rates, over all proteins.

To obtain ω we first write (14) as

$$W(x) = \frac{x}{F - Bx}. \tag{97}$$

Its inverse becomes

$$W^{-1}(x) = \frac{Fx}{1 + Bx}, \tag{98}$$

which, with $M_2(x) = x$ (96), expands using (53) as

$$M_2\big(W^{-1}(x)\big) = \frac{Fx}{1 + Bx} \sim x - x^2 + O(x^3). \tag{99}$$

Here the leading powers are $\Gamma(0) = 1$ and $\Gamma(1) = 2$. supplementary equation (56) predicts that

$$\omega = 1 - \Gamma(0) = 0 \tag{100}$$

and (79)

$$\xi = -1, \tag{101}$$

as supported by the results shown in the main paper (Figs. 2 and 3 in main text).

Regulatory dynamics $\mathbb{R}$

In gene regulation a transcription factor j impacts gene i, when its protein X_j binds to gene i's promoter site in the DNA. The bound X_j can either activate i to produce X_i or inhibit its production, depending on the regulatory relationship between i and j. As only a limited number of X_j proteins can be bound to the promoter site at any given time, the impact of j on i reaches saturation in the limit of a large X_j population. Hence, regulatory dynamics are often modeled using a saturating *switch-like* function, which approaches unity for *e.g.*, activation. A most common choice is a Hill function of the form [13, 14]

$$\mathcal{H}(x) = \frac{x^h}{1 + x^h}, \tag{102}$$

which satisfies $\mathcal{H}(x \to \infty) \to 1$. The Hill coefficient h controls the rate of the saturation, being gradual for small h and rapid for large h, approaching a step-function in the limit $h \to \infty$. Using (102) we model gene regulation via

$$\frac{dx_i(t)}{dt} = -Bx_i(t) + \sum_{j=1}^{N} A_{ij}\mathcal{H}(x_j(t)), \tag{103}$$

where the first term accounts for protein degradation, and the second term describes i's activation by all its interacting partners. Hence in this model we have

$$\begin{aligned} M_0(x_i(t)) &= -Bx_i(t) \\ M_1(x_i(t)) &= 1 \\ M_2(x_j(t)) &= \mathcal{H}(x_j(t)). \end{aligned} \tag{104}$$

Here $W(x)$ (14) satisfies

$$W(x) \sim W^{-1}(x) \sim \frac{1}{x}, \tag{105}$$

which, in $M_2(x)$ (104) leads to

$$M_2\big(W^{-1}(x)\big) = \mathcal{H}\left(\frac{1}{x}\right) \sim \frac{1}{1 + x^h}. \tag{106}$$

To obtain ω we expand (106) following (53), obtaining

$$M_2\big(W^{-1}(x)\big) \sim 1 - x^h + x^{2h} + O(x^{3h}), \tag{107}$$

hence $\Gamma(0) = 0$ and $\Gamma(1) = h$, providing (56)

$$\omega = 1 - \Gamma(1) = 1 - h \tag{108}$$

and (79)

$$\xi = -h. \tag{109}$$

In our results we used two versions of regulatory models: $\mathbb{R}_1$ with $h = \frac{1}{3}$ and $\mathbb{R}_2$ with $h = 2$. Hence for $\mathbb{R}_1$ we predict $\omega = \frac{2}{3}$ and $\xi = -\frac{1}{3}$, and for $\mathbb{R}_2$ we predict $\omega = -1$ and $\xi = -2$, both predictions in agreement with the results presented in the main paper (Figs. 2 and 3).

Mutualistic dynamics $\mathbb{M}$

We consider symbiotic eco-systems, such as plant-pollinator networks, in which different species exhibit mutualistic interactions. The species populations follow the dynamic equation

$$\frac{dx_i(t)}{dt} = Bx_i(t)\left(1 - \frac{x_i(t)}{C}\right) + \sum_{j=1}^{N} A_{ij}x_i(t)F\big(x_j(t)\big). \tag{110}$$

The first term on the r.h.s.,

$$M_0(x) = Bx\left(1 - \frac{x}{C}\right) \tag{111}$$

captures logistic growth: when the population is small, the species reproduces at a rate B, yet, as x_i approaches the carrying capacity of the system C, growth is hindered by competition over limited resources [15]. The mutualistic interactions are captured by

$$\begin{aligned} M_1(x) &= x \\ M_2(x) &= F(x), \end{aligned} \tag{112}$$

where $F(x)$ represents the functional response, describing the positive impact that species j has on species i. This functional response can take one of several forms [16]:

Type I: linear impact

$$F(x) = \alpha x. \tag{113}$$

Type II: saturating impact [16]

$$F(x) = \frac{\alpha x}{1 + \alpha x}. \tag{114}$$

Type III: A generalization of Type II, where [17]

$$F(x) = \frac{\alpha x^h}{1 + \alpha x^h}. \tag{115}$$

In our simulations we used Type III mutualistic interactions, providing

$$\begin{aligned} M_0(x) &= Bx\left(1 - \frac{x}{C}\right) \\ M_1(x) &= x \\ M_2(x) &= \frac{\alpha x^h}{1 + \alpha x^h} \end{aligned} . \tag{116}$$

where we set $B = C = 1$. Hence we have (14)

$$W(x) = -\frac{1}{1 - x}, \tag{117}$$

and therefore

$$W^{-1}(x) \sim \frac{1 - x}{x}. \tag{118}$$

To obtain ω we use (53) and take $M_2(x)$ from (116). We obtain

$$M_2\big(W^{-1}(x)\big) = \frac{\alpha\left(\frac{1-x}{x}\right)^h}{1 + \alpha\left(\frac{1-x}{x}\right)^h}, \tag{119}$$

which, in leading terms of x becomes

$$M_2\big(W^{-1}(x)\big) \sim 1 - x^h + x^{2h} + O(x^{3h}), \tag{120}$$

whose leading powers are $\Gamma(0) = 0$ and $\Gamma(1) = h$. Hence supplementary equations (56) and (79) predict

$$\omega = 1 - \Gamma(0) = 1 - h, \tag{121}$$

and

$$\xi = -h. \tag{122}$$

Choosing $h = 2$ in the main text we find, indeed, that $\omega = -1$, a degree-averting dynamics, and $\xi = -2$, (Figs. 2 and 3 in main paper).

Population dynamics $\mathbb{P}$

Birth-death processes have many applications in population dynamics [18], queuing theory [19] or biology [18]. We consider a network in which the nodes represent sites, each site i having a population $x_i(t)$, with population diffusion enabled between neighboring sites. This process can be described by a dynamic equation of the form

$$\frac{\mathrm{d}x_i(t)}{\mathrm{d}t} = -Bx_i^b(t) + \sum_{j=1}^{N} A_{ij} x_j^a(t). \tag{123}$$

The first term on the r.h.s. represents the internal dynamics of site i, characterized by the exponent b. In queuing dynamics, choosing $b = 0$ represents a constant influx (out-flux) into (out of) site i; in population dynamics mortality can be represented by setting $b = 1$, indicating that the number of deaths per unit time is proportional to the current population at i. The second term describes the flow from i's neighboring sites j into i, which is often linear in x_j, namely $a = 1$. Here, to test the limits of our theory we select a rather extreme pair of exponents $a = 2$ and $b = 3$, which, as we show below, lead to a relatively large ω. This choice provides

$$W(x) \sim x^{-3}, \tag{124}$$

$$W^{-1}(x) \sim x^{-\frac{1}{3}} \tag{125}$$

and hence

$$M_2\big(W^{-1}(x)\big) \sim x^{-\frac{2}{3}}. \tag{126}$$

The leading powers in this expansion are simply $\Gamma(0) = -2/3$ (with no additional powers in the series), for which supplementary equation (56) predicts

$$\omega = 1 - \Gamma(0) = \frac{5}{3} \tag{127}$$

and

$$\xi = \frac{2}{3}, \tag{128}$$

a rather extreme example of degree-driven flow (Figs. 2 and 3 in main paper).

Dynamics	Equation	Symbol	ω	ξ	Class
**Population**	$\frac{dx_i(t)}{dt} = -x_i^3(t) + \sum_{j=1}^{N} A_{ij} x_i^2(t)$	$\mathbb{P}$	$\frac{5}{3}$	$\frac{2}{3}$	**Degree Driven**
**Regulatory**	$\frac{dx_i(t)}{dt} = -x_i(t) + \sum_{j=1}^{N} A_{ij} \frac{x_j^{\frac{1}{3}}(t)}{1 + x_j^{\frac{1}{3}}(t)}$	$\mathbb{R}_1$	$\frac{2}{3}$	$-\frac{1}{3}$	**Degree Driven**
**Epidemic**	$\frac{dx_i(t)}{dt} = -x_i(t) + \sum_{j=1}^{N} A_{ij} \big(1 - x_i(t)\big) x_j(t)$	$\mathbb{E}$	0	-1	**Homogeneous**
**Biochemical**	$\frac{dx_i(t)}{dt} = 1 - x_i(t) - \sum_{j=1}^{N} A_{ij} x_i(t) x_j(t)$	$\mathbb{B}$	0	-1	**Homogeneous**
**Mutualistic**	$\frac{dx_i(t)}{dt} = x_i(t)\big(1 - x_i(t)\big) + \sum_{j=1}^{N} A_{ij} x_i(t) \frac{x_j^2(t)}{1 + x_j^2(t)}$	$\mathbb{M}$	-1	-2	**Degree Avert**
**Regulatory**	$\frac{dx_i(t)}{dt} = -x_i(t) + \sum_{j=1}^{N} A_{ij} \frac{x_j^2(t)}{1 + x_j^2(t)}$	$\mathbb{R}_2$	-1	-2	**Degree Avert**

Supplementary Table 1: **Dynamic models - summary.** We tested the flow patterns of six different dynamic systems, from gene regulation to epidemic spread. For each system we present the relevant dynamic equation, the exponents ω and ξ, and the resulting dynamic classification.

Supplementary Note 3
Methods and data analysis

Numerical simulation

To numerically test our predictions we used a fourth-order Runge-Kutta stepper (Matlab's ode45) to solve each of the models in Table 1. Starting from an arbitrary initial condition we allowed the system to reach its steady state, applying the termination condition

$$\max_{i=1}^{N} \left| \frac{x_i(t_n) - x_i(t_{n-1})}{x_i(t_n)\Delta t_n} \right| < \varepsilon, \tag{129}$$

where t_n is the time stamp of the nth Runge-Kutta step and $\Delta t_n = t_n - t_{n-1}$. As the system approaches the steady-state, the activities $x_i(t_n)$ become almost independent of time, and the numerical derivative $x_i(t_n) - x_i(t_{n-1})/x_i(t_n)\Delta t_n \approx \dot{x}_i/x_i$ approaches zero. The condition (129) guarantees that the maximum of $\dot{x}_i/x_i$ over all activities $x_i(t_n)$ is smaller than a pre-defined termination variable ε. In our simulations we set $\varepsilon \leq 10^{-11}$, a rather strict condition, to ensure that our system is sufficiently close to the true steady state.

Measuring G

To obtain the response matrix G_{mn} (9) we set the initial condition of the system at $x_m(t = 0) = x_m$, namely we begin with the system at the steady-state (as described above). We then perturb a single node n by setting its initial condition to be

$$x_n(t = 0) = (1 + \alpha)x_n, \tag{130}$$

representing signal of magnitude $\mathrm{d}x_n = \alpha x_n$. We set $\alpha = -0.1$, simulating a perturbation of -10% in n's activity. Such negative perturbation ($\alpha < 0$) ensures that in case the activities are bounded, such as in $\mathbb{E}$, where $0 \leq x_n \leq 1$, our perturbation does not drive x_n to the restricted $x_n > 1$ zone. The perturbation (130) will force the system away from its initial steady state, leading to the perturbed

state $x_m(t \to \infty) = x_m + \mathrm{d}x_m$, $(m = 1, \ldots, N,\ m \neq n)$. To obtain this perturbed state we numerically solve

$$\begin{cases} \dfrac{\mathrm{d}x_n}{\mathrm{d}t} & = & 0 \\ & & \\ \dfrac{\mathrm{d}x_m}{\mathrm{d}t} & = & M_0(x_m) + \sum_{q=1}^{N} A_{mq} M_1(x_m) M_2(x_q) \quad m \neq n \end{cases}, \tag{131}$$

in which the perturbation on n is held constant in time, and the remaining $N-1$ nodes respond via supplementary equation (1). The perturbed steady state is reached when (131) satisfied the condition (129), namely when all $N-1$ nodes have reached their new steady state. The nth column of the response matrix (9) is then

$$G_{mn} = \left| \frac{\dfrac{x_m(t \to \infty) - x_m(t=0)}{x_m(t=0)}}{\dfrac{x_n(t \to \infty) - x_n(t=0)}{x_n(t=0)}} \right| = \left| \frac{\dfrac{\mathrm{d}x_m}{x_m}}{\dfrac{\mathrm{d}x_n}{x_n}} \right| = \left| \frac{\mathrm{d}x_m}{\alpha x_m} \right| \tag{132}$$

where we used $\mathrm{d}x_{m(n)}$ to represent the change in node $m(n)$'s state due to the perturbation. In the last step above we used (130) to write $\mathrm{d}x_n/x_n = \alpha$. Repeating this process for all $n = 1, \ldots, N$ source nodes we obtain numerically all columns of G_{mn}.

Measuring $\mathcal{F}_i$

To obtain the flow we must calculate $G_{mn}^{\{i\}}$ in (32), representing the response matrix G_{mn} under the silencing of i. The m, n term of this matrix is calculated by following a similar procedure to calculating G_{mn} above, only here we constrain $x_i(t)$ to remain at its original steady-state, blocking its contribution to the flow of information from n to m. This is achieved by solving

$$\begin{cases} \frac{\mathrm{d}x_n}{\mathrm{d}t} &= 0 \\ \frac{\mathrm{d}x_i}{\mathrm{d}t} &= 0 \\ \frac{\mathrm{d}x_m}{\mathrm{d}t} &= M_0(x_m) + \sum_{q=1}^{N} A_{mq} M_1(x_m) M_2(x_q) \quad m \neq i, n \end{cases}, \tag{133}$$

hence $x_i(t) = x_i$ remains unperturbed, $x_n(t) = (1+\alpha)x_n$, is subject to a permanent perturbation, and the remaining $N-2$ nodes converge to their perturbed state $x_m(t \to \infty) = x_m(t=0) + \mathrm{d}x_m^{\{i\}}$. As above, the nth column of $G^{\{i\}}$ becomes

$$G_{mn}^{\{i\}} = \frac{\dfrac{x_m(t \to \infty) - x_m(t=0)}{x_m(t=0)}}{\dfrac{x_n(t \to \infty) - x_n(t=0)}{x_n(t=0)}} = \frac{\mathrm{d}x_m^{\{i\}}}{\alpha x_m}. \tag{134}$$

Once we obtain $G_{mn}^{\{i\}}$ numerically, we use (32) to extract $\mathcal{F}_{mn}^{\{i\}}$, (33) to obtain $\mathcal{F}_n^{\{i\}}$ and finally, (34) to calculate the desired $\mathcal{F}_i$.

Scalable calculation of $\mathcal{F}_i$. The calcalation outlined above is a direct numerical realization of $\mathcal{F}_i$ which is highly accurate, but also unscalable. Indeed, to obtain the flow through a single node one must solve a set of N equations N times, *i.e.* solving (133) for all nodes $n = 1, \ldots, N$. If we wish to calculate $\mathcal{F}_i$ for all $i = 1, \ldots, N$ nodes, the numerical calculation becomes unfeasible. The computational burden of solving N coupled equations scales as $O(N^3)$, hence for N nodes, each requiring N repetitive solutions we come up with $O(N^5)$, a prohibitive computation for networks of order $N = 10^3$ to 10^4 nodes. To overcome this limitation we seek a method to evaluate $\mathcal{F}_i$, without the need to repetitively calculate $G_{mn}^{\{i\}}$ for all $i = 1, \ldots, N$. This can be achieved using supplementary equation (38), which allows us to express $G_{mn}^{\{i\}}$ directly from G_{mn}, using a linear approximation, as

$$G_{mn}^{\{i\}} \approx G_{mn} - G_{mi} G_{in}, \tag{135}$$

exact in the limit of small perturbations. Therefore, in our numerical calculation of $\mathcal{F}_i$ we calculated G_{mn} only once, using (132), and then approximated $G_{mn}^{\{i\}}$ directly

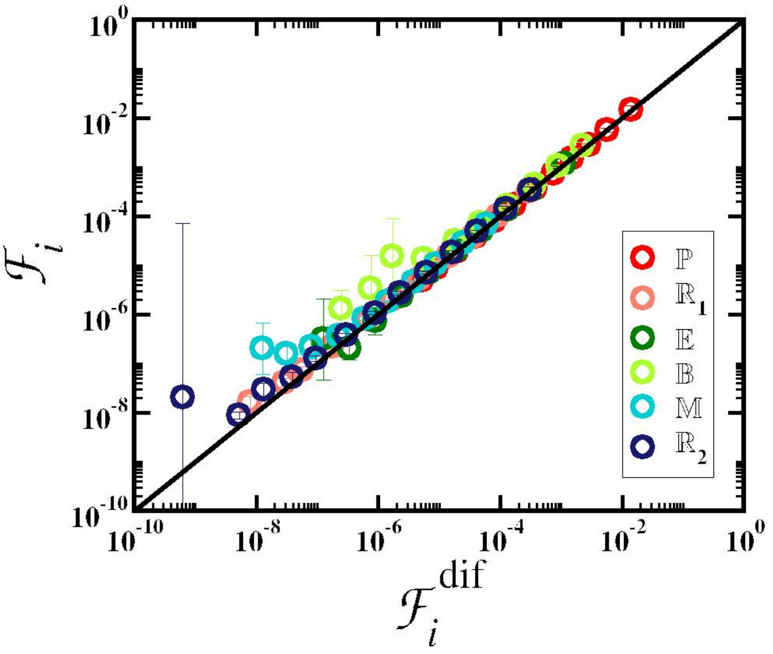

Supplementary Figure 3: **Approximating $\mathcal{F}_i$.** To evaluate the approximation of (135) we compared it with the exact calculation (134), using a directed scale-free network of $N = 500$ nodes, on which we implemented each of the six dynamic models of Table 1. We find that the results condense tightly around $y = x$ for over eight orders of magnitude (solid black line), confirming the validity of the approximate (135). In our remaining results, obtained for larger networks ($N \sim 10^3 - 10^4$), the exact calculation becomes unscalable, hence we use the approximate (135) instead. Error bars represent 95% confidence intervals (Supplementary Note 3)

from (135). This allows us to evaluate $\mathcal{F}_i$ by solving the N equations of (133) only N times, instead of N^2. To test the validity of this calculation we constructed a meso-scale network of $N = 500$ nodes, with a scale-free degree distribution ($P(k) \sim k^{-\gamma}$, $\gamma = 3$). Thanks to the smaller scale of this network, we are able to calculate $\mathcal{F}_i$ exactly using (134). We then compared the results of this exact solution to the approximate (135), for each of the six models of Supplementary Table 1. We show in Supplementary Figure. 3 that our approximate calculation agrees with the exact (134), confirming the validity of our scalable scheme for obtaining $\mathcal{F}_i$

Measuring $\mathcal{F}_{ij}$

To obtain $\mathcal{F}_{ij}$ (61) we first calculate $G_{mn}^{\{ij\}}$ in (60), namely the response matrix G_{mn} under the silencing of the i,j edge. Note that in our notation the directed edge A_{ij} represents an edge outgoing from j and incoming to i, namely $A_{ij} = A_{i \leftarrow j}$. Indeed in the dynamic equation (1) A_{ij} quantifies the rate by which x_j impacts $\mathrm{d}x_i/\,\mathrm{d}t$ capturing information flowing from j to i. Hence $G_{mn}^{\{ij\}}$ represents the flow of information from n to m, under the silencing of information flow from j to i. To understand the meaning of such silencing, consider a perturbation on node n, $x_n \to x_n + \mathrm{d}x_n$. The information from this perturbation reaches the target node i through all of i's nearest incoming neighbors, among which is the node j. Silencing the link A_{ij} terminates all information flow from the perturbed n to the target i through j. It does maintain, however, the flow to i from its other incoming neighbors. Also note that in this process node i continues to receive input from j's unperturbed state x_j, it just does not "sense" j's perturbation $\mathrm{d}x_j$. To realize all this we set the initial condition of all nodes to their the steady state, $x_m(t=0) = x_m$, except for the perturbed n, for which, as before

$$x_n(t=0) = (1+\alpha)x_n. \tag{136}$$

We then solve the A_{ij} silenced equations

$$\begin{cases} \dfrac{\mathrm{d}x_n}{\mathrm{d}t} &= 0 \\[2ex] \dfrac{\mathrm{d}x_i}{\mathrm{d}t} &= M_0\big(x_i(t)\big) + \displaystyle\sum_{\substack{q=1\\q\neq j}}^{N} A_{iq} M_1\big(x_i(t)\big) M_2\big(x_q(t)\big) + A_{ij} M_1\big(x_i(t)\big) M_2(x_j) \\[2ex] \dfrac{\mathrm{d}x_m}{\mathrm{d}t} &= M_0\big(x_m(t)\big) + \displaystyle\sum_{q=1}^{N} A_{mq} M_1\big(x_m(t)\big) M_2\big(x_q(t)\big) \qquad m \neq i,n \end{cases}, \tag{137}$$

where the time-independent x_j at the r.h.s. of the second equation (as opposed to $x_j(t)$) represents the unperturbed steady state of node j. The first equation in (137) ensures the permanent perturbation on the source node n and the last

equation captures the response of all nodes $m = 1, \ldots, N$ to this perturbation. For node i we allow information to flow through all nodes except for j, hence the interaction term (sum on r.h.s. of second equation) excludes node j, and an additional term is added, where i is exposed to j's unperturbed state x_j (third term on r.h.s. of second equation). Note, that here we do not silence the node j, rather we silence the information flow from that node to i, hence we only freeze j's state in the appropriate interaction term in the ith equation, but do allow x_j to respond to $\mathrm{d}x_n$ in all remaining equations.

For an undirected network, silencing the i, j edge amounts to freezing the flow through both A_{ij} and A_{ji}, an effective silencing of two reciprocal edges. In supplementary equation (137) this maps to adding an additional edge silenced equation for node j as

$$\frac{\mathrm{d}x_j}{\mathrm{d}t} = M_0\big(x_j(t)\big) + \sum_{\substack{q=1 \\ q \neq i}}^{N} A_{jq} M_1\big(x_j(t)\big) M_2\big(x_q(t)\big) + A_{ji} M_1\big(x_j(t)\big) M_2(x_i), \quad (138)$$

effectively sileneing two edges, A_{ij} in the ith equation and A_{ji} in the jth equation.

After reaching the steady state, supplementary equation (137) (together with (138) if required) provides us with the silenced $G_{mn}^{\{ij\}}$ via

$$G_{mn}^{\{ij\}} = \left| \frac{\dfrac{x_m(t \to \infty) - x_m(t = 0)}{x_m(t = 0)}}{\dfrac{x_n(t \to \infty) - x_n(t = 0)}{x_n(t = 0)}} \right| = \left| \frac{\mathrm{d}x_m^{\{ij\}}}{\alpha x_m} \right|, \quad (139)$$

capturing the response of all nodes m to the n perturbation under the termination of information flow through A_{ij}. Using (139) in (61), and taking G_{ij} from (132) we can directly calculate $\mathcal{F}_{ij}$. As explained in Sec. 3 direct calculation of $\mathcal{F}_{ij}$ becomes unscalable for large networks. Hence in our results we used (66) to approximate $\mathcal{F}_{ij}$ directly from G_{ij} (132). Using the fact that for nearest neighbor nodes $R_{mn} \approx G_{mn}$, namely the local (nearest neighbor) response is almost fully determined by the direct m, n interaction, we further approximate (66) by

$$\mathcal{F}_{ij} \approx G_{ji} \left[\frac{1}{N} \sum_{n=1}^{N} \left(\frac{\sum_{m=1}^{N} G_{in} G_{mj}}{\sum_{m=1}^{N} G_{mn}} \right) \right], \tag{140}$$

allowing to calculate $\mathcal{F}_{ij}$ directly from the numerically obtained G_{mn} (132). Hence in all the results presented in the main text we first solved for G using (131) and (132), and then evaluated $\mathcal{F}_{ij}$ from (140).

Network construction

We used a diverse set of model and real networks on which to test our predictions. The model networks include both weighted and unweighted networks with both random and scale-free topologies, as outlined below.

Model networks

We generated four types of model networks:
Erdős-Rényi (ER). A binary random graph with $N = 6,000$ nodes; each pair of nodes linked independently with probability $p = 2 \times 10^{-3}$, resulting in $L = 35,765$ undirected links and an average degree of $\langle k \rangle = 11.9$.
Scale-free. We constructed three types of scale free networks. (i) **Binary (SF3)** - An unweighted scale-free network with $6,000$ nodes and $L = 23,994$ links, which we constructed using the Barabási-Albert model [20]. The network features a scale-free degree distribution $P(k) \sim k^{-\gamma}$ with $\gamma = 3$. Below we denote this unweighted topology by T_{ij}. (ii) **Normal weights (SF4)** - Using T_{ij} we introduced normally distributed weights W_{ij} on all existing links ($T_{ij} \neq 0$). The result is a weighted scale-free network, $A_{ij} = T_{ij} W_{ij}$, where the weights W_{ij} are extracted from $W_{ij} \sim N(\mu, \sigma^2)$, a normal distribution with mean $\mu = 10$ and variance $\sigma^2 = 9$. (iii) **Scale-free weights (SF1)** - Here we extracted the weights W_{ij} from a scale-free probability density function $P(w) \sim w^{-\alpha}$, $\alpha = 3$, resulting in $A_{ij} = T_{ij} W_{ij}$ which has both a scale-free topology and a scale-free weight distribution. (iv) **Directed (SF2)** - to construct a directed network we used the Barabási-Albert model [20] to construct two scale-free networks, T_{ij}^{in} and T_{ij}^{out}. In the former, each new node draws $m = 5$ incoming links to the already existing nodes, and in the

latter it draws $m = 5$ out-going links from the existing nodes. The results is a pair of independent directed networks, one with a scale-free distribution of in-degrees (T_{ij}^{in}) and the other with a scale-free distribution of out-degrees (T_{ij}^{out}). We then used the sum of the two networks $T_{ij}^{\text{Dir}} = T_{ij}^{\text{in}} + T_{ij}^{\text{out}}$ to construct a directed scale-free network with both the in-degree and the out-degree following independent power law distributions. Also here we generated the weights W_{ij} from a scale-free probability density function $P(w) \sim w^{-\alpha}$, $\alpha = 3$, resulting in $A_{ij}^{\text{Dir}} = T_{ij}^{\text{Dir}} W_{ij}$ which has independent scale-free in-degree and out-degree topology coupled with scale-free weights.

Empirical networks

In addition to the four model networks we also tested our predictions on a set of empirically constructed networks, ranging from sub-cellular biology to online social networks. On each network we implemented the appropriate type of dynamics.

Email Epoch (SIS model $\mathbb{E}$). We used the Email Epoch dataset [21], recording 3×10^5 emails sent between $3,188$ individuals over the course of $T = 83$ days. We constructed a weighted social network, in which A_{ij} equals the overall volume of emails that j sent to i. This results in an asymmetric network $A_{ij} \neq A_{ji}$, with a giant connected component of $N = 3,185$ nodes and $L = 63,710$ weighted links. Both the degree and the weight distributions are fat-tailed, representing a high level of degree/weight heterogeneity.

UCIonline (SIS model $\mathbb{E}$). An instant messaging network from the University of California Irvine [22], capturing $\sim 6 \times 10^4$ transactions between $1,899$ users during a $T = 218$ day period. We used the number of messages sent between each pair of nodes to evaluate the weights A_{ij}, obtaining again an asymmetric network whose giant connected component has $N = 1,893$ nodes and $L = 27,670$ weighted links. Similarly to Email Epoch, UCIonline also exhibits fat-tailed degree/weight distributions.

HumanPPI (Biochemical dynamics $\mathbb{B}$). The Human protein protein interaction (PPI) network, a scale-free network, consisting of $N = 3,125$ nodes (protein types) and $L = 13,854$ undirected links, representing chemical interactions between protein types.

YeastPPI (Biochemical dynamics $\mathbb{B}$). A set of $L = 5,036$ links, capturing the chemical interactions between $N = 1,647$ proteins (nodes) in yeast [23]. These protein interactions comprise an undirected network with a scale-free degree distribution.

Ecological networks Eco1 and Eco2 (Mutualistic dynamics $\mathbb{M}$). To construct mutualistic networks we collected data on symbiotic interactions between plants and pollinators in the Carlinville, Illinois eco-system (https://www.nceas.ucsb.edu/interactionweb/html/robertson_1929.html). The network consists of 456 plant species that are linked to one or more of the 1,429 pollinators, forming a bipartite network, M_{ik} where $i = 1, \dots, 456$ represents plant species, and $k = 1, \dots, 1,429$ represents pollinators. By projecting the bipartite network on the plant/pollinator set we construct two networks: the 456×456 plant network A_{ij} and the $1,429 \times 1,429$ pollinator network B_{ij}, in which the nodes are linked by mutualistic interactions. Indeed, if two plants i and j are pollinated by the same pollinator k they mutually benefit each other, since each of them contributes to k's abundance, and hence, indirectly to each others pollination. The weight of the i, j interaction is determined by the density of mutual symbiotic relationships between i and j: (i) the more mutual pollinators k that plants i and j share the stronger the mutualistic interaction between them; (ii) on the other hand the more plants pollinated by k the smaller is its contribution to each plant. Hence we estimate the contribution of each mutual pollinator k to the link weight between plants i and j as

$$\frac{M_{ik} M_{jk}}{\sum_{s=1}^{n} M_{sk}}, \tag{141}$$

where the denominator accounts for the overall pollination capacity of k. This results in the weighted plant network

$$A_{ij} = \sum_{k=1}^{m} \frac{M_{ik} M_{jk}}{\sum_{s=1}^{n} M_{sk}} \tag{142}$$

and by a similar procedure, the weighted pollinator network [24]

$$B_{kj} = \sum_{i=1}^{n} \frac{M_{ik} M_{ij}}{\sum_{s=1}^{m} M_{is}}. \tag{143}$$

In this process it is possible to have isolated components, *e.g*, single disconnected nodes. The state of these isolated nodes is decoupled from the state of the rest of the network, and hence in our analysis we only focused on the giant connected component of A_{ij} and B_{ij}. For A_{ij} this giant component includes all nodes (456), and for B_{ij} it includes a total of 1,044 out of the 1,429 pollinators in the eco-system.

Logarithmic binning

To analyze the scaling properties of $\mathcal{F}_i$ and $\mathcal{F}_{ij}$ we used logarithmic binning [25]. Consider the function $y_m(x_m)$, a noisy function which on average scales as $y_i(x_i) \sim x_i^\alpha$, (*e.g*, $\mathcal{F}_m \sim S_m^\omega$, $m = 1, \ldots, N$). To properly test this relationship we first divide all data-points m into W bins as

$$\mathbb{M}(w) = \{m : c^{w-1} < x_m \leq c^w\}, \tag{144}$$

where $w = 1, \ldots, W$ and c is a constant. In (144) the wth bin includes all data-points m whose x_m is between c^{w-1} and c^w. The parameter c is selected such that the unity of all bins $\bigcup_{w=1}^W \mathbb{M}(w)$ includes all data-points, hence we set $c^W = \max x_m$. We then plot the average x_i of the data-points in each bin $x_w = \langle x_m \rangle_{m \in \mathbb{M}(w)}$ versus the average value of the function y_i in that bin $y(x_w) = \langle y_m \rangle_{m \in \mathbb{M}(w)}$. For instance, to plot $\mathcal{F}_m$ vs. S_m for SF1 in Supplementary Figure. 3 of the main paper, we divided all nodes into $W = 11$ bins, the first, $\mathbb{M}(1)$, consisting of all nodes with $0 < S_i < 3.43$; the second, $\mathbb{M}(2)$, with $3.43 < S_i < 5.58$, until we reached the last bin, $\mathbb{M}(11)$, which had $436.91 < S_i < 575.7$ (575.7 being the maximal weighted degree). For each bin we plot the average flow $\langle \mathcal{F}_m \rangle$ in that bin vs. the average degree $\langle S_m \rangle$. To estimate the error in each bin we uses Cox method [26] which captures the 95% confidence interval for log-normal distributed data.

Supplementary Note 4
Additional validation

Our analytical derivations, outlined in Secs. 1 and 2 are exact under two main assumptions: (i) the perturbative limit of small signals $\mathrm{d}x$, which allows us to use linear response theoretic tools; (ii) the configuration model [4] pertaining to A_{ij}, according to which node i's nearest neighbor statistics are independent of i. In real scenarios we are often confronted by large perturbations, or by empirical networks, which may violate, to some extent, the clean picture of the configuration model. Therefore we tested the robustness of our analytically predicted scaling, (55) and (58), against deviations from assumptions (i) and (ii) above. Specifically, regarding (i), we test the impact of large perturbations. Regarding (ii) we introduce two topological features that are frequently observed in real networks, but violate the configuration model framework: degree-degree correlations and clustering. These non-local topological characteristics are a fingerprint of non-random connectivity, overriding the essential ingredient of the configuration model.

Large perturbation

Measuring $\mathcal{F}_i$ entails introducing a signal, $\mathrm{d}x_n$, to the steady state activity x_n of the source node n, and observing the patterns of information flow from n to all remaining nodes through the intermediate node i. Throughout the paper we set the magnitude of our signals to 10% of the source's steady state, namely $\alpha = \mathrm{d}x_n/x_n = 0.1$. In Supplementary Figure. 4 we examine the impact of larger perturbations, setting $\alpha = 0.5$, a 50% perturbation, and $\alpha = 0.8$, a large perturbation of 80%. We find that the predicted scaling ω is extremely robust, largely unaffected by the size of the initial perturbation. This lack of sensitivity is rooted in the well-established robustness of scaling relationships, which are often unaffected by small deviations and discrepancies [27]. This is especially relevant in a scale free environment, where the steady state activities x_n are broadly distributed, often spanning orders of magnitude. Under these conditions a small perturbation is one that does not change the order of magnitude of x_n, namely one where $\log \alpha$ is small, rather than

		Network	Degree Correlations Q	Clustering C
Empirical		ECO1	0.007	0.1739
		ECO2	0.005	0.1397
		Human PPI	-0.2234	0.0437
		Yeast PPI	-0.1024	0.1908
		UCIonline	0.0418	0.2639
		Email Epoch	0.083	0.041
Model networks	C	SF3	-0.04	0.008
		C_1	-0.028	0.0504
		C_2	0.0183	0.1004
		C_3	0.2184	0.1524
	Q	Q_1	0.101	0.0005
		Q_2	0.2	0.0003
		Q_3	0.3	0.0004
		Q_4	0.4	0.0008
		Q_5	0.5	0.0005

Supplementary Table 2: **Deviations from the configuration model**. The clustering C and the degree correlations Q of our set of empirical networks. Many of them are shown to exhibit relatively high C and Q, in violation of the configuration model approximation, and yet, despite that, our theoretical results seem to be unaffected, indicating the robustness of our predictions. We also used SF3 to construct additional networks with controlled levels of C and Q, allowing us to further investigate the impact of these topological characteristics. The source network SF3, a random scale-free network, has negligible C and Q (top row of Model Networks). Then, through rewiring we introduced increasing levels of clustering C_1 - C_3, and degree correlations Q_1 - Q_5. (The rewiring process for clustering also induces degree correlations.)

α. Indeed, in a scale free environment, the nearest neighbors of x_n are not too sensitive to perturbations in x_n, as long as x_n's order of magnitude remains the same. Hence we find that the linear response framework remains valid even under unambiguously large perturbations.

The effect of clustering

Next we consider the impact of clustering C, representing the network's tendency to from triads, in which there is an increased probability for an n, m link, if n and m share a mutual neighbor i. Under the configuration model assumption, clustering tends to zero if the network is sparse and $N \to \infty$ [4]. Most empirical networks, however, feature non vanishing levels of clustering, in some cases reaching an order of $C \sim 10^{-1}$ [28]. To measure node i's clustering we write (in an undirected network)

$$C_i = \frac{\sum_{m,n=1}^{N} A_{im} A_{in} A_{nm}}{\binom{k_i}{2}}, \tag{145}$$

in which the numerator counts the number of actual triads involving nearest neighbors of i, and the denominator equals to the number of possible triads around i, *i.e.* the number of potential pairs among i's k_i nearest neighbors. Hence $0 \le C_i \le 1$ is the fraction of potential triads that are actually present among i's neighbors. The clustering of the network is then obtained by averaging over all nodes as

$$C = \frac{1}{N} \sum_{i=1}^{N} C_i. \tag{146}$$

In Table 2 we show the clustering C as obtained from our set of model and empirical networks. We find that for some of these networks C is rather high, in some cases reaching as much as $C = 0.2639$ (UCIonline). Still, as demonstrated in the main text, our analytical predictions performed well, even under these challenging conditions of extreme clustering. This indicates that our predictions are robust against empirically observed levels of clustering. To further examine the effects of clustering in a controlled fashion, we used the scale-free network SF3, and gradually increased its clustering to $C_1 = 0.05, C_2 = 0.1$ and $C_3 = 0.15$ (Table 2). We then measured $\mathcal{F}_i$ vs. S_i on each of these networks. We find, in this setting, which is controlled for C, that clustering does have a minor effect on the flow, observing that extreme levels of clustering systematically diminish the role of the hubs, shifting the flow curve below the predicted scaling, Supplementary Figure 5.

The origins of this discrepancy lie in the fact that in the presence of triads (large C), highly connected nodes are typically surrounded by many triadic loops. As a result most node pairs, whose shortest path traverses through a hub, can now be linked by several alternative paths of comparable length. These multiple pathways (or loops) undermine the role of the hubs as information mediators, effectively reducing the value of the exponent ω. For instance, for $\mathbb{R}_1$ we predict $\omega = 2/3$, but when clustering becomes dominant we find that the actual slope is slightly lower (Supplementary Figure. 5b, triangles). Similar trends are also observed for $\mathbb{E}$, $\mathbb{M}$ and $\mathbb{R}_2$ (Supplementary Figure. 5c,e,f). Note, that even if ω is slightly lower than predicted, say $w \approx -1.2$ instead of $\omega = -1$, as observed for $\mathbb{M}$ and $\mathbb{R}_2$), the qualitative prediction, that these two models exhibit degree-averting flow, remains valid. Hence, despite minor discrepancies in our quantitative prediction pertaining to the values of ω and ξ, the essential insight on the system's large scale flow patterns - degree driven (red), homogeneous (green) or degree-averting (blue) - is robust also under high C levels.

The observed effect of the mildly decreased ω is rooted in purely topological grounds: the enrichment of loops, providing multiple pathways for information flow to bypass the hubs. Therefore it is independent of the dynamics, as confirmed by the consistent reduction in hub-flow in both degree driven, homogeneous and degree averting systems. This provides us with a qualitative assessment, that C slightly diminishes ω, pushing the system to be less degree-driven, an effect that is observed under all dynamic regimes.

The effect of degree-degree correlations

Next we examine the effect of degree correlations Q, as defined in Ref. [5]. As before, we first observe the correlation levels exhibited by our set of empirical networks, finding that the two biological networks, Human PPI and Yeast PPI, have strongly negative degree correlations (Table 2). The fact that our predictions cover these two networks is, once again, an indication of our theory's robustness against empirically observed correlations. To complement this finding we used SF3 and rewired it to obtain networks of increasing levels of (positive) degree correlations. As in the case of clustering, here too we find that positive degree

correlations undermine the role of the hubs (Supplementary Figure. 6). Indeed, under $Q > 0$, hubs tend to link to other highly connected nodes. Therefore, even if we freeze a hub, blocking its contribution to the flow, other hub/s in its vicinity open alternative pathways for information flow, reducing each individual hub's flow-centrality. Note that the results we present in Supplementary Figure. 6 go as high as $Q = 0.4$, a rather extreme level of degree correlations, that significantly exceeds the levels practically observed in most empirical networks [5].

Predictability limits of our theory - quantitative vs. qualitative. Our theory provides both quantitative as well as qualitative predictions. At the quantitative level, we predict the precise value of ω and ξ, allowing us to provide accurate assessments of the contribution of all nodes/links to the flow. No less important are, however, our qualitative predictions, that allow us to translate ω and ξ into direct insights on the macroscopic flow patterns of a networked system, distinguishing between the degree-driven, homogeneous or degree averting dynamic universality classes. While the precise value of ω and ξ may, in some cases, be sensitive to the network's fine structure, *e.g.*, C or Q, its macro-scale behavior is extremely robust, sensitive only to the intrinsic mechanisms of the system's inner dynamics. For instance, the degree driven $\mathbb{R}_1$ may exhibit a slight decrease in ω due to clustering, but such micro and meso-scopic discrepancies cannot cause a qualitative shift to a different class, turning, for instance from degree-driven to degree-averting. Such transition can only be done by altering the system's internal mechanisms, such as shifting from $\mathbb{R}_1$ ($\omega = 2/3$) to $\mathbb{R}_2$ ($\omega = -1$), a change in the physics of the node interactions, which requires a fundamental intervention, unattainable by minor discrepancies. Therefore our qualitative predictions and classifications are highly robust against such deviations in our model assumptions, even under challenging conditions where our quantitative predictions may show observable deviations.

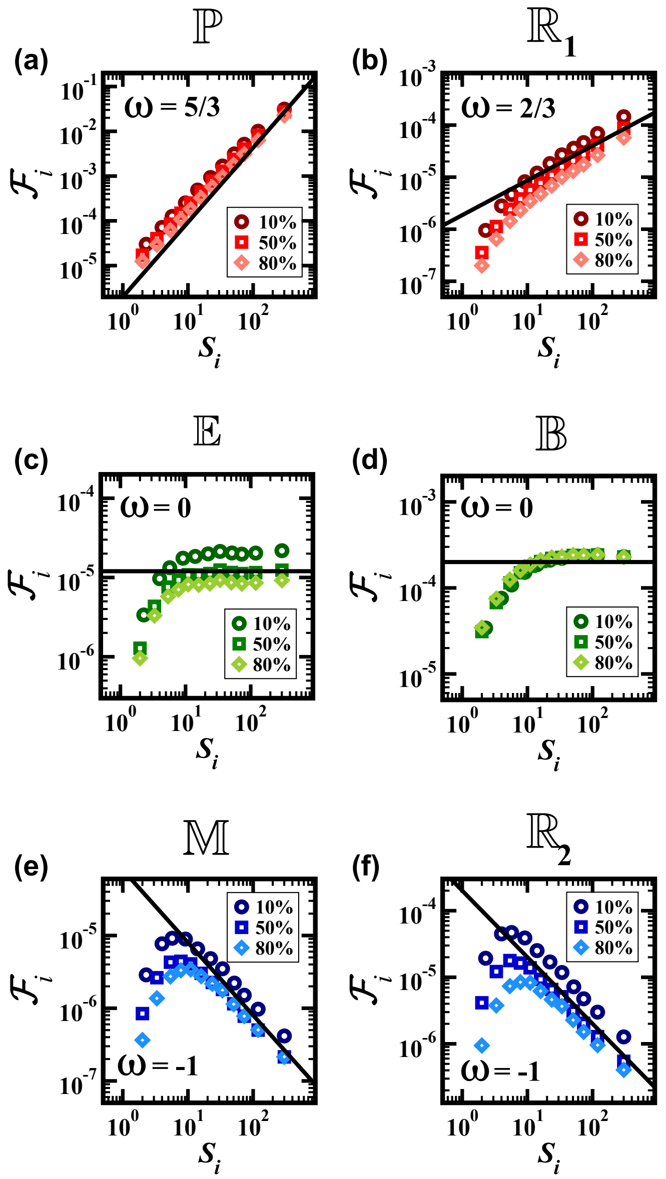

Supplementary Figure 4: **The effect of perturbation size**. To test the limits of our linear response framework we measured the flow $\mathcal{F}_i$ vs. S_i , as obtained for large signals, representing an $\alpha = 10\%$ (circles), 50% (squares) and 80% (diamonds) perturbation. We find that the perturbation size has no visible effect on the macroscopic patterns of flow, with ω consistently adhering to the predicted value (solid lines). The dynamic models are (a) Population dynamics $\mathbb{P}$; (b) Regulatory dynamics $\mathbb{R}_1$; (c) the SIS model $\mathbb{E}$; (d) Biochemical dynamics $\mathbb{B}$; (e) Mutualsim $\mathbb{M}$ and (f) our other Regulatory model $\mathbb{R}_2$ (see Table 1).

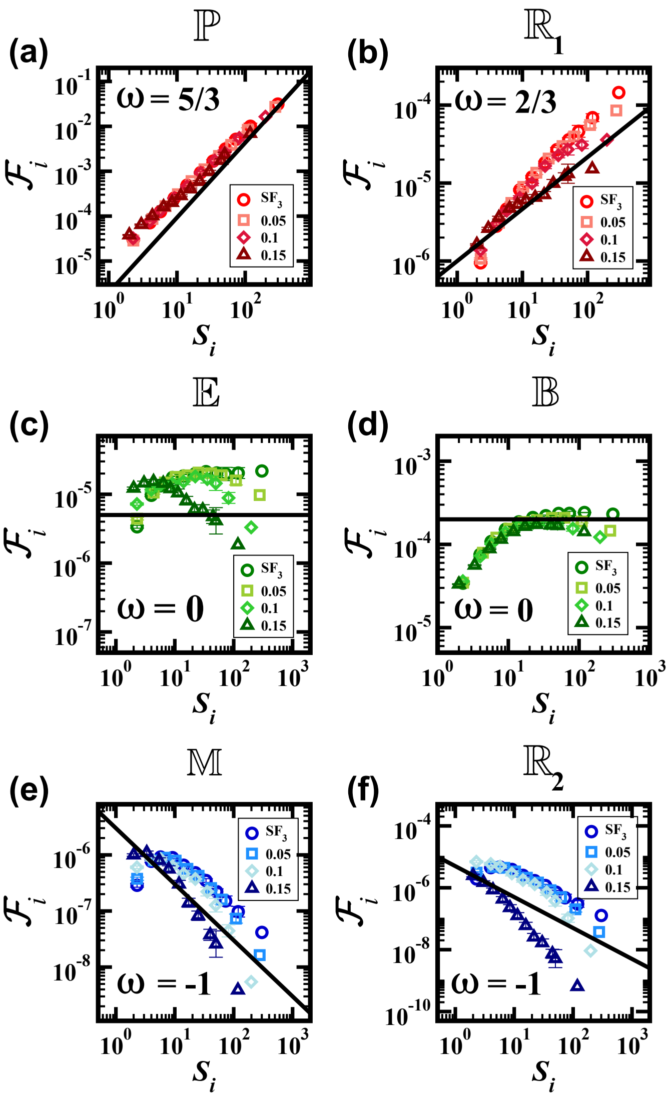

Supplementary Figure 5: **The effect of clustering** C. We used SF3 and rewired it to introduce increasing levels of clustering $C = 0.05, 0.1, 0.15$. We find that for high clustering (triangles) the impact of the hubs on the flow is diminished, effectively leading to a slight decrease in the value of ω. The effect is similar regardless of the dynamics ($\mathbb{P}, \mathbb{R}_1, \dots$), albeit most dramatic in $\mathbb{E}, \mathbb{M}$ and $\mathbb{R}_2$. The dynamic models are (a) Population dynamics $\mathbb{P}$; (b) Regulatory dynamics $\mathbb{R}_1$; (c) the SIS model $\mathbb{E}$; (d) Biochemical dynamics $\mathbb{B}$; (e) Mutualsim $\mathbb{M}$ and (f) our other Regulatory model $\mathbb{R}_2$ (see Table 1). Error bars represent 95% confidence intervals (Supplementary Note 3)

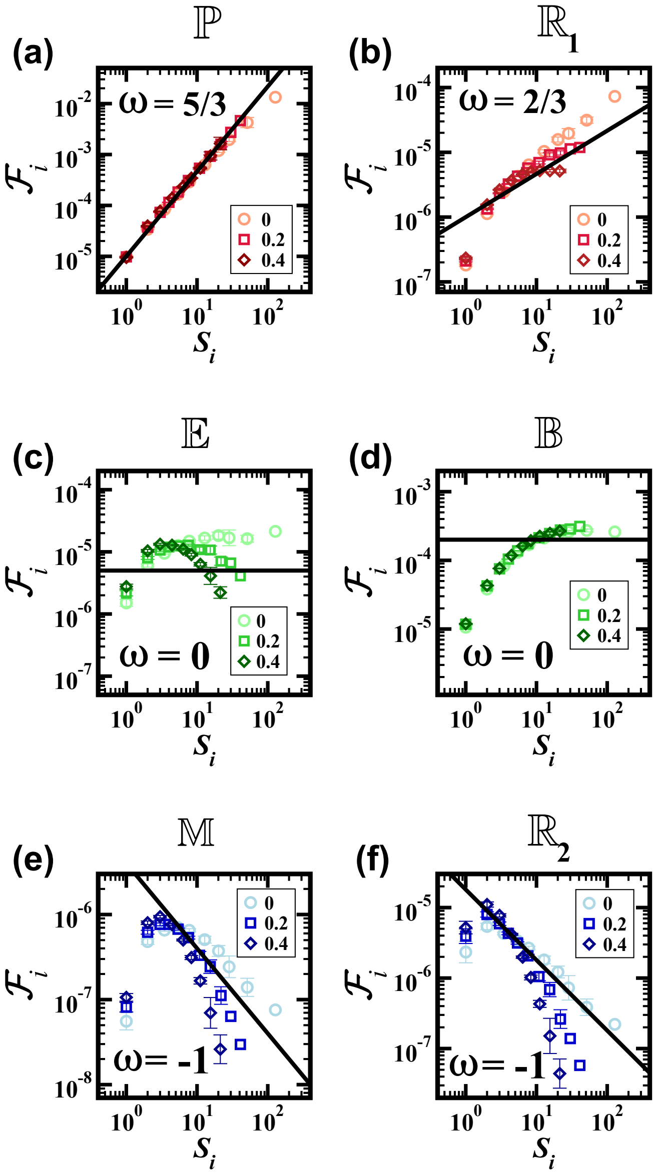

Supplementary Figure 6: **The effect of degree-correlations** Q. We used SF3 and rewired it to introduce increasing levels of degree-correlations $Q = 0.2, 0.4$, the latter exceeding the practically observed levels of correlations in empirical networks [5]. We find that for high Q (diamonds) the impact of the hubs on the flow is, generally, diminished, effectively leading to a decrease in the observed value of ω. The dynamic models are (a) Population dynamics $\mathbb{P}$; (b) Regulatory dynamics $\mathbb{R}_1$; (c) the SIS model $\mathbb{E}$; (d) Biochemical dynamics $\mathbb{B}$; (e) Mutualsim $\mathbb{M}$ and (f) our other Regulatory model $\mathbb{R}_2$ (see Table 1). Error bars represent 95% confidence intervals(Supplementary Note 3)

Supplementary Note 5
Epidemic spread via air-traffic

To follow the patterns of flow during the spread of an epidemic we used the susceptible-infected-recovered (SIR) model [6, 8]. In this framework nodes are either susceptible (S), infected (I) or recovered (R), where recovered nodes can no longer receive or transmit the disease (immune or dead). The dynamic processes can be summarized by

$$I + S \xrightarrow{\rho} 2I \tag{147}$$

$$I \xrightarrow{\beta} R, \tag{148}$$

capturing the infection process, followed by the recovery, in which infected nodes (I) irreversibly transition to recovered (R). These processes translate to the coupled dynamic equations

$$\frac{\mathrm{d}S_i}{\mathrm{d}t} = -\rho \sum_{j=1}^{N} A_{ij} S_i I_j \tag{149}$$

$$\frac{\mathrm{d}I_i}{\mathrm{d}t} = -\beta I_i + \rho \sum_{j=1}^{N} A_{ij} S_i I_j \tag{150}$$

$$\frac{\mathrm{d}R_i}{\mathrm{d}t} = \beta I_i, \tag{151}$$

where $S_i(t) + I_i(t) + R_i(t) = 1$, represent the probability of individual i (or fraction of population i) to be susceptible, infected or recovered, respectively. At the healthy state we have $S_i = 1$ and $I_i = R_i = 0$ for all i. When an outbreak emerges at some arbitrary source node a spreading process is initiated, reaching at $t \to \infty$ a state where $I_i = 0$ and the remaining population is split among recovered individuals, who were impacted by the disease, and susceptible individuals, who were never infected. Hence, in this context, we define the spread of information

from the local outbreak to the rest of the network as

$$Z = \sum_{m=1}^{\infty} R_m(t \to \infty), \tag{152}$$

capturing the overall response of the system through the total number of infected individuals.

To evaluate the contribution of a specific node i to Z, we freeze its activity at time t_0, setting the i-equations in (149) - (151) to

$$\begin{aligned} \left.\frac{\mathrm{d}S_i}{\mathrm{d}t}\right|_{t \geq t_0} &= 0 \\ \left.\frac{\mathrm{d}I_i}{\mathrm{d}t}\right|_{t \geq t_0} &= -BI_i \end{aligned} \tag{153}$$

therefore prohibiting any future infections in i, and gradually transitioning all current i infections to the recovered state. The remaining equations for all other nodes in (149) - (151) remain unchanged. This is equivalent to immunization of i, which changes all current susceptible individuals at t_0 into an immune state, effectively eliminating the infection process in the equations for S_i and I_i in (149) and (150). Such freezing of i will impact the overall response of the system, diminishing Z to

$$Z^{\{i,t_0\}} = \sum_{m=1}^{N} R_m^{\{i,t_0\}}(t \to \infty), \tag{154}$$

which quantifies the recovered population within all nodes under i's immunization at $t = t_0$. Taken together i's time-dependent contribution to the flow is therefore captured by

$$\mathcal{F}_i(t_0) = \frac{Z - Z^{\{i,t_0\}}}{Z}, \tag{155}$$

the fractional change in the number of individuals impacted by the disease due to i's immunization (or freezing) at t_0. Our results in Fig. 4 of the main text show that for $t_0 \to 0$, $\mathcal{F}_i(t_0) \sim S_i$, namely it is best to immunize the hubs; however as t_0 advances, the contribution of the hubs to the flow decreases, until $\mathcal{F}_i(t_0 \to \infty)$ exhibits degree-averting flow.

Numerical simulation

To construct the empirical air-traffic network we collected data from the Official Airline Guide [29], comprising human mobility information of over 7×10^6 daily passenger exchanges between $N = 1,292$ airports, representing the $\sim 30\%$ busiest airports worldwide, which together account for $\sim 80\%$ of all air-travel. The data aggregates three years worth of travel, namely $\sim 8 \times 10^9$ entries in which a single passenger traveled from airport j to airport i. This allowed us to construct the weighted and directed network F_{ij}, whose terms equal to the average number of daily passengers departing from j, arriving at i. To construct the SIR equations (149) - (151) we track the number of infected and susceptible individuals in each location, focusing on the absolute numbers of susceptible/infected/recovered individuals in i, namely

$$\mathcal{S}_i(t) = n_i S_i(t) \tag{156}$$

$$\mathcal{I}_i(t) = n_i I_i(t) \tag{157}$$

$$\mathcal{R}_i(t) = n_i R_i(t), \tag{158}$$

where n_i is the populations size of i, hence $\mathcal{S}_i(t) + \mathcal{I}_i(t) + \mathcal{R}_i(t) = n_i$. Using this notation, the SIR equations become

$$\frac{\mathrm{d}\mathcal{S}_i}{\mathrm{d}t} = -\rho \sum_{j=1}^{N} \frac{F_{ij}}{n_j} \mathcal{S}_i \mathcal{I}_j \tag{159}$$

$$\frac{\mathrm{d}\mathcal{I}_i}{\mathrm{d}t} = -\beta \mathcal{I}_i + \rho \sum_{j=1}^{N} \frac{F_{ij}}{n_j} \mathcal{S}_i \mathcal{I}_j \tag{160}$$

$$\frac{\mathrm{d}\mathcal{R}_i}{\mathrm{d}t} = \beta \mathcal{I}_i. \tag{161}$$

Here β is the recovery rate per individual per unit time (day), typically of order 10^{-1} to 1, capturing the fact that most infections last for a few days to several weeks, and ρ determines the reproduction rate of the spreading disease, capturing the probability, per pair of individuals at the same location, to result in a new infection. The coupling F_{ij}/n_j is the daily probability of a randomly selected

individual from j to visit i. Finally, $\mathcal{S}_i\mathcal{I}_j$ represents the total number of pairs of i-susceptible and j-infected individuals at time t. Therefore we can express the infection rate in (159) by rearranging it as

$$\mathcal{I}_j \frac{F_{ij}}{n_j}\rho\mathcal{S}_i = \begin{pmatrix}\text{Number of} \\ \text{infected } j \\ \text{individuals}\end{pmatrix}\begin{pmatrix}\text{Probability} \\ \text{for a } j \text{ indi-} \\ \text{vidual to fly} \\ \text{to } i \text{ today}\end{pmatrix}\begin{pmatrix}\text{Infections} \\ \text{that he/she} \\ \text{causes in } i\end{pmatrix}\begin{pmatrix}\text{Number of} \\ \text{susceptibles} \\ \text{in } i\end{pmatrix}. \tag{162}$$

Next we divide all equations in (159) - (161) by n_i, to obtain normalized equations of the form (149) - (151), in which S_i, I_i and R_i capture the fraction of susceptible/infected/recovered in each population. The result is precisely the SIR equations above, in which the relevant network is $A_{ij} = F_{ij}$, namely A_{ij} is the average number of individuals traveling daily from j to i. In principle A_{ij} may be different from A_{ji}, however, in practice we find that they are almost identical, as, indeed, the vast majority of human travel is bidirectional. Therefore, we simplify our network to be [29]

$$A_{ij} = \frac{F_{ij} + F_{ji}}{2}, \tag{163}$$

a symmetric weighted network, with rather extreme levels of topological and weight heterogeneity ($P(k) \sim k^{-1.2}, P(w)$ fat-tailed, albeit not power-law). The weighted degree S_i in this network, represents the daily incoming passenger volume, which is, on average $\langle S \rangle \approx 3 \times 10^3$ for this network. For a disease to become pandemic, *i.e.* spread efficiently at a global scale, we must tune the infection rate ρ, such that this number of individuals entering i generates a viable spread. Hence we set

$$\beta = 0.1 \tag{164}$$

$$\rho = 0.5 \times 10^{-4}, \tag{165}$$

which produces a reproduction rate $R_0 = \rho\langle S^2 \rangle / \beta\langle S \rangle > 1$, ensuring a globally spreading epidemic [30].

To obtain the results presented in Fig. 4 of the main text we set the system, initially, at the healthy state $S_i(t=0)=1$, $I_i(t=0)=R_i(t=0)=0$ for all i. We then generated an outbreak at the Addis-Ababa airport (ADD, $i=1$), setting $S_1(t=0)=0.999, I_1(t=0)=10^{-3}, R_1(t=0)=0$, and tracked the spread in time by numerically solving (149) - (151) with the parameters taken from (163) to (165). To extract the flow we froze each of the nodes $i=1,\dots,1,292$, by freezing their instantaneous $S_i(t)$ as appears in (153), repeating this process in three selected time-points $t=0,3$ and 10. Hence, we solved the SIR equations a total of $1,292\times 3$ times, to obtain the flow through all nodes for each time-point.

Supplementary Note 6

Flow analysis of the Glycolysis pathway

We consider Glycolysis, a fundamental metabolic pathway, that regulates the chemical transformation of glucose to ATP [31]. The process consists of a set of 10 chemical reactions between 18 metabolites, catalyzed by 10 proteins, together comprising $N = 28$ nodes, as illustrated in Supplementary Figure. 7. We use mass-action-kinetics to track the concentration of all reacting components in the system. Each chemical reaction is captured by a stoichiometric equation of the form

$$\sum_{i=1}^{N} a_i X_i \xrightarrow{k_{\vec{a}}} \sum_{i=1}^{N} b_i X_i, \tag{166}$$

in which a combination of a_i reactants of type X_i on the l.h.s. produce the products on the r.h.s. at a rate $k_{\vec{a}}$. The vector $\vec{a} = (a_1, \dots, a_N)$ represents the stoichiometric coefficients of each reaction, a typically sparse vector, with a few entries of $a_i = 1$ or 2, depicting the fact that most reactions are of low order, involving only a few interacting molecules X_i. Each reaction is represented by a network module, denoted by $\vec{a}$, in which all the reactants i whose $a_i \neq 0$ are linked. These modules are represented in Supplementary Figure. 7 by the large grey circles that contain all reacting nodes (small circles, representing interactants). Hence we have, together, 28 nodes (i) for all reacting molecules (X_i) that are grouped in 10 modules, representing all reactions ($\vec{a}$).

The outgoing flux from the $\vec{a}$ module is proportional to the reaction rate $k_{\vec{a}}$ and to the abundance of combinations including a_i copies of X_i molecules. Following the the law of mass action this provides the module flux as [32, 33]

$$F_{\vec{a}} = k_{\vec{a}} \prod_{i=1}^{N} \frac{x_i^{a_i}}{a_i!}, \tag{167}$$

where $x_i = x_i(t)$ is the instantaneous concentration of the X_i reactant. This flux, outgoing from module $\vec{a}$ produces the products on the r.h.s. of (166), as represented in Supplementary Figure. 7 by the arrows leading from modules to product nodes.

Hence, the incoming flux into each product on the r.h.s. of (166) is

$$F_{\vec{a}\to i} = k_{\vec{a}} b_i \prod_{i=1}^{N} \frac{x_i^{a_i}}{a_i!}, \tag{168}$$

where the b_i factor captures the fact that each reaction $\vec{a}$, produces b_i copies of the product X_i. We can now write the system's dynamic equations for each node i as

$$\frac{\mathrm{d}x_i}{\mathrm{d}t} = \sum_{\vec{a}} \Big(F_{\vec{a}\to i} - a_i F_{\vec{a}} \Big), \tag{169}$$

a summation over the incoming flux to i from all modules, balanced by a depletion of a_i copies of X_i, as the reactants of module $\vec{a}$ interact at rate $F_{\vec{a}}$.

To construct the complete Glycolysis kinetic equations we use the following additional guidelines

- Reversible reactions of the form $\sum_i a_i X_i \leftrightharpoons \sum_i b_i X_i$ are counted as two separate reactions from left to right, once placing the module $\vec{a}$ at the l.h.s. and once placing $\vec{b}$ at the l.h.s..

- Molecules with no incoming flux, *i.e.* that are not formed by any of the Glycolysis reactions, are assigned a constant influx k_i, hence they are introduced to the pathway from an external source. This is equivalent, formally, to adding the effective reaction $\emptyset \xrightarrow{k_i} X_i$, which, according to (167) creates a constant influx with rate k_i incoming to X_i. There are three such out-sourced molecules in the Glycolysis pathway, Glucose, NAD^+ and Pi. To this we add the protein catalysts (nodes $1, \dots, 10$), which are synthesized by the DNA at constant rate k_i.

- All 28 nodes are depleted from the system via degradation at rate b_i, a self-reaction of the form $X_i \xrightarrow{b_i} \emptyset$, contributing a linear term $-b_i x_i(t)$ to each of the mass-action equations $i = 1, \dots, 28$.

- All modules include a protein (indexed $1, \dots, 10$), which acts as a catalyst for the reaction. This protein is not consumed by the reaction, and becomes available again one the reaction is completed. However, a bound protein, namely one that is in the process of catalysis is temporarily depleted from

the system, until the reaction is completed and it becomes available again. To account for this we define 10 additional nodes, $i = 29, \ldots, 38$, representing bound complexes of catalyst and reactants. Each reaction becomes a two step reaction: Module $\leftrightharpoons$ Bound complex $\to$ Products. The bound complexes are treated as additional nodes in the kinetic equations.

From reactions to equation terms. As an example we consider the first reaction, represented by the upper left module in Supplementary Figure. 7, consisting of the nodes 1 (protein catalyst), 11 (Glucose) and 22 (ATP). This module represents the chain reaction

$$X_1 + X_{11} + X_{22} \underset{k_r^{(1)}}{\overset{k_f^{(1)}}{\rightleftharpoons}} X_{29} \overset{k_{\mathrm{cat}}^{(1)}}{\to} X_1 + X_{12} + X_{23}, \tag{170}$$

in which protein 1 catalyzes the reaction between 11 and 22, to form the bound complex 29. The latter can then either dissociate back to 11 and 12 at rate $k_r^{(1)}$, or produce 12 and 23 at a rate $k_{\mathrm{cat}}^{(1)}$, in each case also freeing again the protein 1. This reaction contributes the following terms to the equations of the reactants. Reactants X_{11} and X_{22} are depleted at rate $k_f^{(1)}$ and replenished by the dissociation of X_{29}:

$$\frac{\mathrm{d}x_{11}}{\mathrm{d}t} = -k_f^{(1)} x_1 x_{11} x_{22} + k_r^{(1)} x_{29} \tag{171}$$

$$\frac{\mathrm{d}x_{22}}{\mathrm{d}t} = -k_f^{(1)} x_1 x_{11} x_{22} + k_r^{(1)} x_{29}. \tag{172}$$

The bound/unbound proteins follow

$$\frac{dx_1}{dt} = -k_f^{(1)} x_1 x_{11} x_{22} + k_r^{(1)} x_{29} + k_{\mathrm{cat}}^{(1)} x_{29} \tag{173}$$

$$\frac{dx_{29}}{dt} = k_f^{(1)} x_1 x_{11} x_{22} - k_r^{(1)} x_{29} - k_{\mathrm{cat}}^{(1)} x_{29}. \tag{174}$$

Finally, the products receive an incoming flux from X_{29}:

$$\frac{dx_{12}}{dt} = k_{\mathrm{cat}}^{(1)} x_{29} \tag{175}$$

$$\frac{dx_{23}}{dt} = k_{\mathrm{cat}}^{(1)} x_{29}. \tag{176}$$

Freezing nodes and modules. To calculate the flow through nodes or mod-

ules we employ the concept of freezing, similar to the way it was defined in the case of pairwise dynamics. Freezing a node X_i entails setting its concentration to the unperturbed steady state value, namely forcing $x_i(t) = x_i$, while allowing the rest of the system to repond to the perturbation. Freezing a module $\vec{a}$ translates to setting the relevant flux $F_{\vec{a}}$ to its unperturbed value. Hence, we solve supplementary equation (169) under the perturbation, allowing the signal to impact all nodes/modules, but replace the flux $F_{\vec{a}}$ associated with the frozen module, by its steady state value, *i.e.* using (167) with $x_i(t) = x_i$ taken at steady state. The meaning is that while all fluxes change, due to concentration changes in the reactants $x_i(t)$, the frozen $F_{\vec{a}}$ remains at its steady state value for all t.

List of reactions and equations

The Glycolysis pathway consists of the following 10 reactions. See Table 3 for indices of all reactants.

$$X_1 + X_{11} + X_{22} \underset{k_r^{(1)}}{\overset{k_f^{(1)}}{\rightleftharpoons}} X_{29} \overset{k_{\text{cat}}^{(1)}}{\rightarrow} X_1 + X_{12} + X_{23} \tag{177}$$

$$X_2 + X_{12} \underset{k_r^{(2)}}{\overset{k_f^{(2)}}{\rightleftharpoons}} X_{30} \overset{k_{\text{cat}}^{(2)}}{\rightarrow} X_2 + X_{13} \tag{178}$$

$$X_3 + X_{13} + X_{22} \underset{k_r^{(3)}}{\overset{k_f^{(3)}}{\rightleftharpoons}} X_{31} \overset{k_{\text{cat}}^{(3)}}{\rightarrow} X_3 + X_{14} + X_{23} \tag{179}$$

$$X_4 + X_{14} \underset{k_r^{(4)}}{\overset{k_f^{(4)}}{\rightleftharpoons}} X_{32} \overset{k_{\text{cat}}^{(4)}}{\rightarrow} X_4 + X_{15} + X_{16} \tag{180}$$

$$X_5 + X_{15} \underset{k_r^{(5)}}{\overset{k_f^{(5)}}{\rightleftharpoons}} X_{33} \overset{k_{\text{cat}}^{(5)}}{\rightarrow} X_5 + X_{16} \tag{181}$$

$$X_6 + X_{16} + X_{25} + X_{26} \underset{k_r^{(6)}}{\overset{k_f^{(6)}}{\rightleftharpoons}} X_{34} \overset{k_{\text{cat}}^{(6)}}{\rightarrow} X_6 + X_{17} + X_{27} + X_{28} \tag{182}$$

$$X_7 + X_{17} + X_{23} \underset{k_r^{(7)}}{\overset{k_f^{(7)}}{\rightleftharpoons}} X_{35} \overset{k_{\text{cat}}^{(7)}}{\rightarrow} X_7 + X_{18} + X_{22} \tag{183}$$

$$X_8 + X_{18} \underset{k_r^{(8)}}{\overset{k_f^{(8)}}{\rightleftharpoons}} X_{36} \overset{k_{\text{cat}}^{(8)}}{\rightarrow} X_8 + X_{19} \tag{184}$$

$$X_9 + X_{19} \underset{k_r^{(9)}}{\overset{k_f^{(9)}}{\rightleftharpoons}} X_{37} \overset{k_{\text{cat}}^{(9)}}{\rightarrow} X_9 + X_{20} + X_{24} \tag{185}$$

$$X_{10} + X_{20} + X_{23} \underset{k_r^{(10)}}{\overset{k_f^{(10)}}{\rightleftharpoons}} X_{38} \overset{k_{\text{cat}}^{(10)}}{\rightarrow} X_{10} + X_{21} + X_{22} \tag{186}$$

Glycolysis mass-action kinetic equations

With 10 catalyst proteins, 18 substrates and 10 bound complexes, supplementary equation (169) comprises a total of 38 coupled equations. Below we include a few representative equations from each class of interacting molecules:

Protein equations

$$\frac{\mathrm{d}x_1}{\mathrm{d}t} = k_1 - b_1 x_1 - k_f^{(1)} x_1 x_{11} x_{22} + k_r^{(1)} x_{29} + k_{\text{cat}}^{(1)} x_{29} \tag{187}$$

$$\frac{\mathrm{d}x_2}{\mathrm{d}t} = k_2 - b_2 x_2 - k_f^{(2)} x_2 x_{12} + k_r^{(2)} x_{30} + k_{\text{cat}}^{(2)} x_{30} \tag{188}$$

$$\frac{\mathrm{d}x_3}{\mathrm{d}t} = k_3 - b_3 x_3 - k_f^{(3)} x_3 x_{13} x_{22} + k_r^{(3)} x_{31} + k_{\text{cat}}^{(3)} x_{31} \tag{189}$$

$$\vdots$$

Note the inclusion of the incoming flux term (k_i), representing protein synthesis, and the degradation term (b_i), which is added to all reactants.

Substrate equations

$$\frac{\mathrm{d}x_{11}}{\mathrm{d}t} = k_{11} - b_{11} x_{11} - k_f^{(1)} x_1 x_{11} x_{22} + k_t^{(1)} x_{29} \tag{190}$$

$$\frac{\mathrm{d}x_{12}}{\mathrm{d}t} = -b_{12} x_{12} + k_{\text{cat}}^{(1)} x_{29} - k_f^{(2)} x_2 x_{12} + k_r^{(2)} x_{30} \tag{191}$$

$$\frac{\mathrm{d}x_{13}}{\mathrm{d}t} = -b_{13} x_{13} + k_{\text{cat}}^{(2)} x_{30} - k_f^{(3)} x_3 x_{13} x_{22} + k_r^{(3)} x_{31} \tag{192}$$

$$\vdots$$

Here the incoming flux term k_{11} appears only in the first equation, representing the fact that the reactant X_{11} (Glucose) is externally introduced into the pathway. Such terms are included also in the equations for X_{25} (NAD^+) and X_{26} (Pi) that are not internally produced in the Glycolysis module. All other substrates lack this term, being produced internally within the Glycolysis interaction sequence.

Bound complex equations

$$\frac{\mathrm{d}x_{29}}{\mathrm{d}t} = k_f^{(1)} x_1 x_{11} x_{22} - k_r^{(1)} x_{29} - k_{\mathrm{cat}}^{(1)} x_{29} \quad (193)$$

$$\frac{\mathrm{d}x_{30}}{\mathrm{d}t} = k_f^{(2)} x_2 x_{12} - k_r^{(2)} x_{30} - k_{\mathrm{cat}}^{(2)} x_{30} \quad (194)$$

$$\frac{\mathrm{d}x_{31}}{\mathrm{d}t} = k_f^{(3)} x_3 x_{13} x_{22} - k_r^{(3)} x_{31} - k_{\mathrm{cat}}^{(3)} x_{31} \quad (195)$$

$$\vdots$$

The bound complex equations lack the degradation term, as these complexes are short lived, serving only as intermediate molecules, depleted only through unbinding (k_r) or catalysis (k_{cat}).

Parameters. In our simulations we used $k_f^{(i)} = k_r^{(i)} = k_{\mathrm{cat}}^{(i)} = 10^{-1}$ ($i = 1, \dots, 38$); $b_i = 10^{-2}$ ($i = 1, \dots, 28$); and where required $k_i = 10^{-2}$ ($i = 1, \dots, 10$ and $i = 11, 25$ and 26).

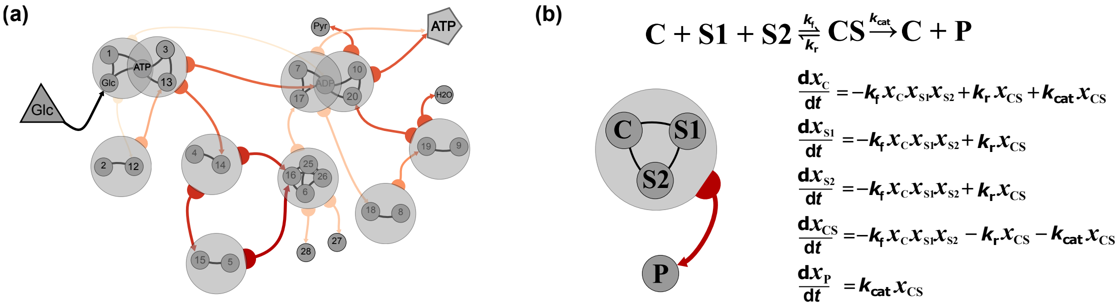

Supplementary Figure 7: **Glycolysis as a modular network**. (a) The Glycolysis pathway consumes glucose (triangle) and produces ATP (pentagon) through a series of 10 chemical reactions of the form (166). Each reaction is symbolized by a module (grey circles) consisting of the interacting molecules, *i.e.* the l.h.s. of 166. The arrows capture the flux outgoing from each module to its products (r.h.s. of (166)), with the flux magnitude represented by the color depth of each arrow. Four isolated nodes represent final products that undergo no further reactions, hence not included in any module. Catalysts, represented by nodes $1, \dots, 10$, are synthesized by the DNA, not through the pathway reactions. Nodes 25 (NAD^+) and 26 (Pi) are also externally introduced into the network, with no incoming flux from the internal network modules. (b) Each interaction module consists of interacting substrates $S1, S2$ and catalyst C, which enables the chemical reaction to produce the product/s P. The catalysis is captured by adding the intermediate species CS, a bound complex of the catalyst and its substrates. We also show the mass-action-kinetic equation terms associated with this reaction.

	Index	Abbreviation	Full name
Catalysts	1	HK	Hexokinase
	2	PGI	Phosphoglucoisomerase
	3	PFK-1	Phosphofructokinase
	4	ALDO	Aldolase
	5	TPI	Triosephosphate
	6	GAPDH	Triose phosphate dehydrogenase
	7	PGK	Phosphoglycerokinase
	8	PGM	Phosphoglyceromutase
	9	ENO	Enolase
	10	PK	Pyruvate kinase
Reactants	11	Glc	Glucose
	12	G6P	Glucose 6-phosphate
	13	F6P	Fructose 6-phosphate
	14	F1,6BP	Fructose 1, 6-bisphosphate
	15	DHAP	Dihydroxyacetone phosphate
	16	GADP	glyceraldehyde 3-phosphate
	17	1,3BPG	1,3-bisphoshoglycerate
	18	3PG	3-Phosphoglycerate
	19	2PG	2-Phosphoglycerate
	20	PEP	phosphoenolpyruvate
	21	Pyr	molecules of pyruvate
	22	ATP	ATP
	23	ADP	ADP
	24	H2O	H2O
	25	NAD+	NAD^+
	26	Pi	Phosphate
	27	NADH	NADH
	28	H+	H^+
Bound complexes	29	HK	Hexokinase
	30	PGI	Phosphoglucoisomerase
	31	PFK-1	Phosphofructokinase
	32	ALDO	Aldolase
	33	TPI	Triosephosphate
	34	GAPDH	Triose phosphate dehydrogenase
	35	PGK	Phosphoglycerokinase
	36	PGM	Phosphoglyceromutase
	37	ENO	Enolase
	38	PK	Pyruvate kinase

Supplementary Table 3: **List of Glycolysis reactants**. The metabolic pathway consists of 10 protein (green, $1, \ldots, 10$), which catalyze the reactions between 18 metabolites (violet, $11, \ldots, 28$). During catalysis the bound proteins (light blue, $29, \ldots, 38$) are modeled effectively as separate nodes in the mass-action kinetic equations.

Supplementary References

[1] B. Barzel and A.-L. Barabási. Universality in network dynamics. *Nature Physics* **9**, 673 (2013).

[2] B. Barzel, Y.-Y. Liu and A.-L. Barabási. Constructing minimal models for complex system dynamics. *Nature communications* **6** (2015).

[3] H. Hahn. Über die nichtarchimedischen Größensysteme. In *Hans Hahn Gesammelte Abhandlungen Band 1/Hans Hahn Collected Works Volume 1*, 445 – 499 (Springer, 1995).

[4] M.E.J. Newman. *Networks - an introduction* (Oxford University Press, New York, 2010).

[5] M.E.J. Newman. Mixing patterns in networks. *Phys. Rev. E* **67**, 026126 (2003).

[6] P.S. Dodds and D.J. Watts. A generalized model of social and biological contagion. *Journal of Theoretical Biology* **232**, 587–604 (2005).

[7] R. Pastor-Satorras and A. Vespignani. Epidemic spreading in scale-free networks. *Phys. Rev. Lett.* **86**, 3200–3203 (2001).

[8] L. Hufnagel, D. Brockmann and T. Geisel. Forecast and control of epidemics in a globalized world. *Proc. Natl. Acad. Sci. USA* **101**, 15124–9 (2004).

[9] E.O. Voit. *Computational Analysis of Biochemical Systems* (Cambridge University Press, New York, NY, 2000).

[10] S. Maslov and I. Ispolatov. Propagation of large concentration changes in reversible protein-binding networks. *Proc. Natl. Acad. Sci. USA* **104**, 13655–60 (2007).

[11] S. Maslov and I. Ispolatov. Spreading out of perturbations in reversible reaction networks. *New Journal of Physics* **9**, 273–283 (2007).

[12] C.W. Gardiner. *Handbook of Stochastic Methods* (Springer-Verlag, Berlin, 2004).

[13] U. Alon. *An Introduction to Systems Biology: Design Principles of Biological Circuits* (Chapman & Hall, London, U.K., 2006).

[14] G. Karlebach and R. Shamir. Modelling and analysis of gene regulatory networks. *Nature Reviews* **9**, 770–780 (2008).

[15] J. Jiang and J. Shi. *Bistability dynamics in structured ecological models* (2009).

[16] C.S. Holling. Some characteristics of simple types of predation and parasitism. *The Canadian Entomologist* **91**, 385–398 (1959).

[17] C.S. Holling. The functional response of predators to prey density and its role in mimicry and population regulation. *Memoirs of the Entomological Society of Canada* **97**, 5–60 (1965).

[18] A.S. Novozhilov, G.P. Karev and E.V. Koonin. Biological applications of the theory of birth-and-death processes. *Briefings in Bioinformatics* **7**, 70–85 (2006).

[19] J.F. Hayes and T.V.J. Ganesh Babu. *Modeling and Analysis of Telecommunications Networks* (John Wiley & Sons, Inc., Hoboken, NJ, USA, 2004).

[20] A.-L. Barabási and R. Albert. Emergence of scaling in random networks. *Science* **286**, 509 (1999).

[21] J.-P. Eckmann, E. Moses and D. Sergi. Entropy of dialogues creates coherent structures in e-mail traffic. *Proc. Natl. Acad. Sci. USA* **101**, 14333–7 (2004).

[22] T. Opsahl and P. Panzarasa. Clustering in weighted networks. *Social Networks* **31**, 155–163 (2009).

[23] H. Yu *et al.* High-quality binary protein interaction map of the yeast interactome network. *Science* **322**, 104110 (2008).

[24] J. Gao, B. Barzel and A.-L. Barabási. Universal resilience patterns in complex networks. *Nature* **530**, 307–312 (2016).

[25] S. Milojević. Power law distributions in information science: Making the case for logarithmic binning. *Journal of the American Society for Information Science and Technology* **61**, 2417–2425 (2010).

[26] X.H. Zhou and S. Gao. Confidence intervals for the log-normal mean. *Statistics in medicine* **16**, 783–790 (1997).

[27] K.G. Wilson. The renormalization group: Critical phenomena and the Kondo problem. *Rev. Mod. Phys.* **47**, 773 (1975).

[28] R. Albert and A.-L. Barabási. Statistical mechanics of complex networks. *Rev. Mod. Phys.* **74**, 47 (2002).

[29] D. Brockmann and D. Helbing. The hidden geometry of complex network-driven contagion. *Science* **342**, 1337 (2013).

[30] R. Pastor-Satorras, C. Castellano, P. Van Mieghem and A. Vespignani. Epidemic processes in complex networks. *Rev. Mod. Phys.* **87**, 925 – 979 (2015).

[31] D.L. Nelson, A.L. Lehninger and M.M.Cox. *Lehninger principles of biochemistry* (Macmillan, 2008).

[32] B. Barzel and O. Biham. Binomial moment equations for stochastic reaction systems. *Physical review letters* **106**, 150602 (2011).

[33] B. Barzel and O. Biham. Stochastic analysis of complex reaction networks using binomial moment equations. *Phys. Rev. E* **86**, 031126 (2012).
