# Supplementary material for: Dynamic patterns of information flow in complex networks
Source: Nat Commun. 2017 Dec 19;8:2181. doi: 10.1038/s41467-017-01916-3 (PMC5736766; doi:10.1038/s41467-017-01916-3)
Supplement: Supplementary file 3 — Description of Additional Supplementary Files [file 41467_2017_1916_MOESM3_ESM.pdf]

Description of Additional Supplementary Files

File Name: Supplementary Software 1
Description: DynamicFlow.m (Matlab) accepts a user defined weighted/directed network A_{ij} and a selected dynamic model $\mathbf{M}$ as input. It provides the flow through all nodes $\mathcal F_i$ and links $\mathcal F_{ij}$, both numerically and theoretically, as in Fig. 3a,b. It also allows to implement the SIR model, as shown in Fig. 5.
