# Supplementary material for: Dynamic patterns of information flow in complex networks
Source: Nat Commun. 2017 Dec 19;8:2181. doi: 10.1038/s41467-017-01916-3 (PMC5736766; doi:10.1038/s41467-017-01916-3)
Supplement: Supplementary file 4 — Supplementary Software 1 [file 41467_2017_1916_MOESM4_ESM.zip › Code/Supplementary code.pdf]

Dynamic patterns of information flow in complex networks

Supplementary code

DynamicFlow.m. The code accepts a user defined network A_{ij}(can be weighed and directed) and dynamics **M**, as input, and provides the dynamic flow patterns as output. Output data is saved in **Results.mat**. Upon initiating, the code prompts you to select between *Models of the form of Eq.*(1) and *SIR model*:

Models of the form of Eq.(1). In this mode the code accepts user inputs on network characteristic and dynamics, and provides as output the node/edge flow $\mathcal{F}_i$ and $\mathcal{F}_{ij}$ (Figs. 3 and 4 in main paper).

<table>
<tr><th>Variable name</th><th colspan="2">Meaning</th></tr>
<tr><td>A</td><td colspan="2">Weighted adjacency matrix</td></tr>
<tr><td>D</td><td colspan="2">Distance matrix</td></tr>
<tr><td>s_in</td><td colspan="2">Weighted in degree</td></tr>
<tr><td>s_out</td><td colspan="2">Weighted out degree</td></tr>
<tr><td rowspan="3">M</td><td>First cell</td><td>M_0</td></tr>
<tr><td>Second cell</td><td>M_1</td></tr>
<tr><td>Third cell</td><td>M_2</td></tr>
<tr><td>G</td><td colspan="2">Response matrix</td></tr>
<tr><td>omega</td><td colspan="2">ω as predicted in Eq. (8)</td></tr>
<tr><td>xi</td><td colspan="2">ξ as predicted in Eq. (9)</td></tr>
<tr><td>Steady_State</td><td colspan="2">Steady state of the system</td></tr>
<tr><td>flow_edge</td><td colspan="2">Edge flow $\mathcal{F}_{ij}$</td></tr>
<tr><td>flow_node</td><td colspan="2">Node flow $\mathcal{F}_i$</td></tr>
</table>

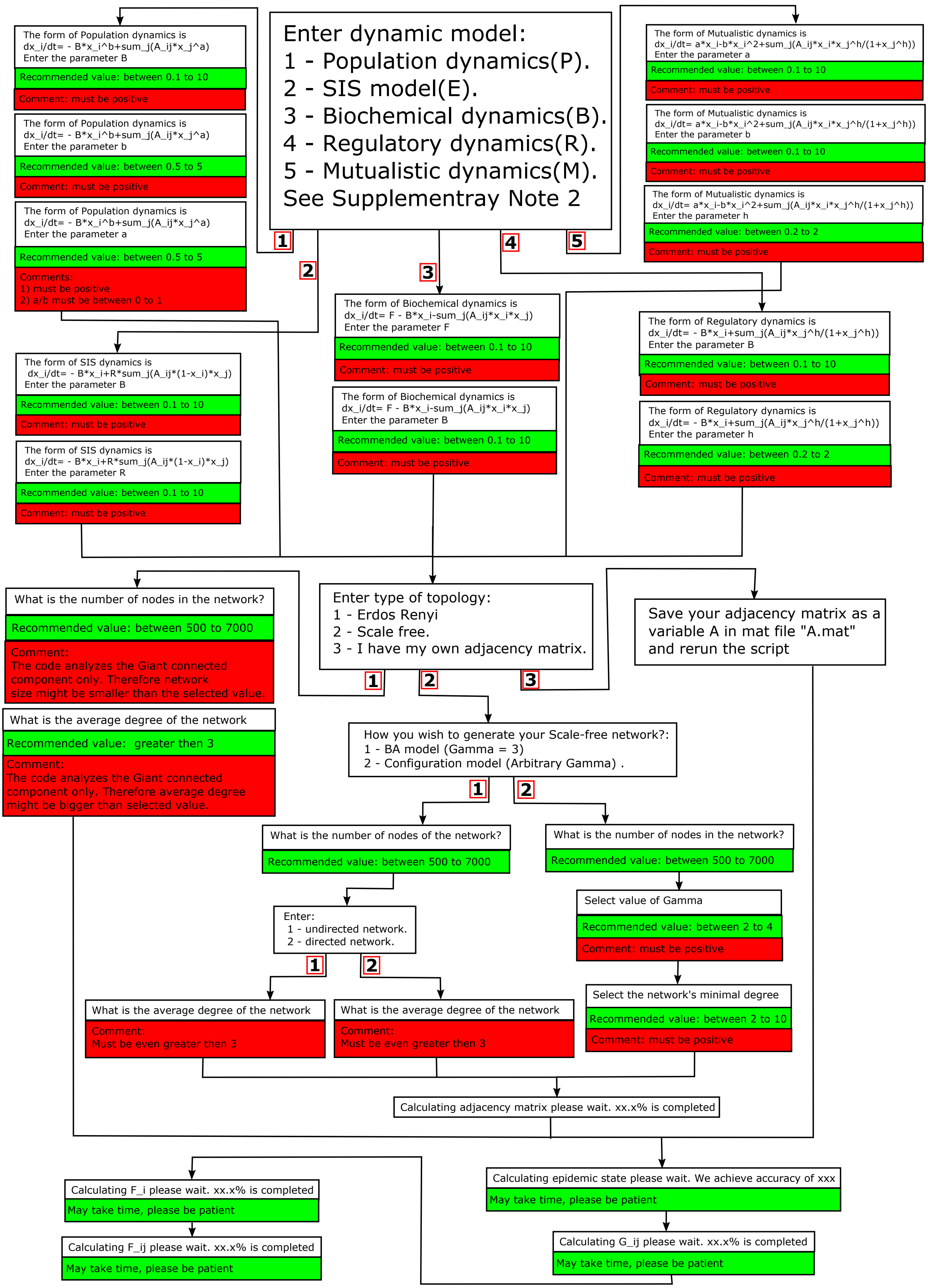

FIG. 1: **User interface for Models of the form of Eq.(1).**

SIR model. In this mode the code accepts a user input network together with the node index representing patient zero, i.e. the location of the initial outbreak. The output provides the flow through all nodes in function of time t_0 (Fig 5 in main paper).

**Variable name**	**Meaning**
A	Weighted adjacency matrix
D	Distance matrix
s_in	Weighted in degree
s_out	Weighted out degree
Steady_State_S	Susceptible steady state
Steady_State_I	Infected steady state
Steady_State_R	Recovered steady state
beta	Beta
rho	Rho
flow	Vector of $\mathcal{F}_i(t_0)$, flow though all node in function of time

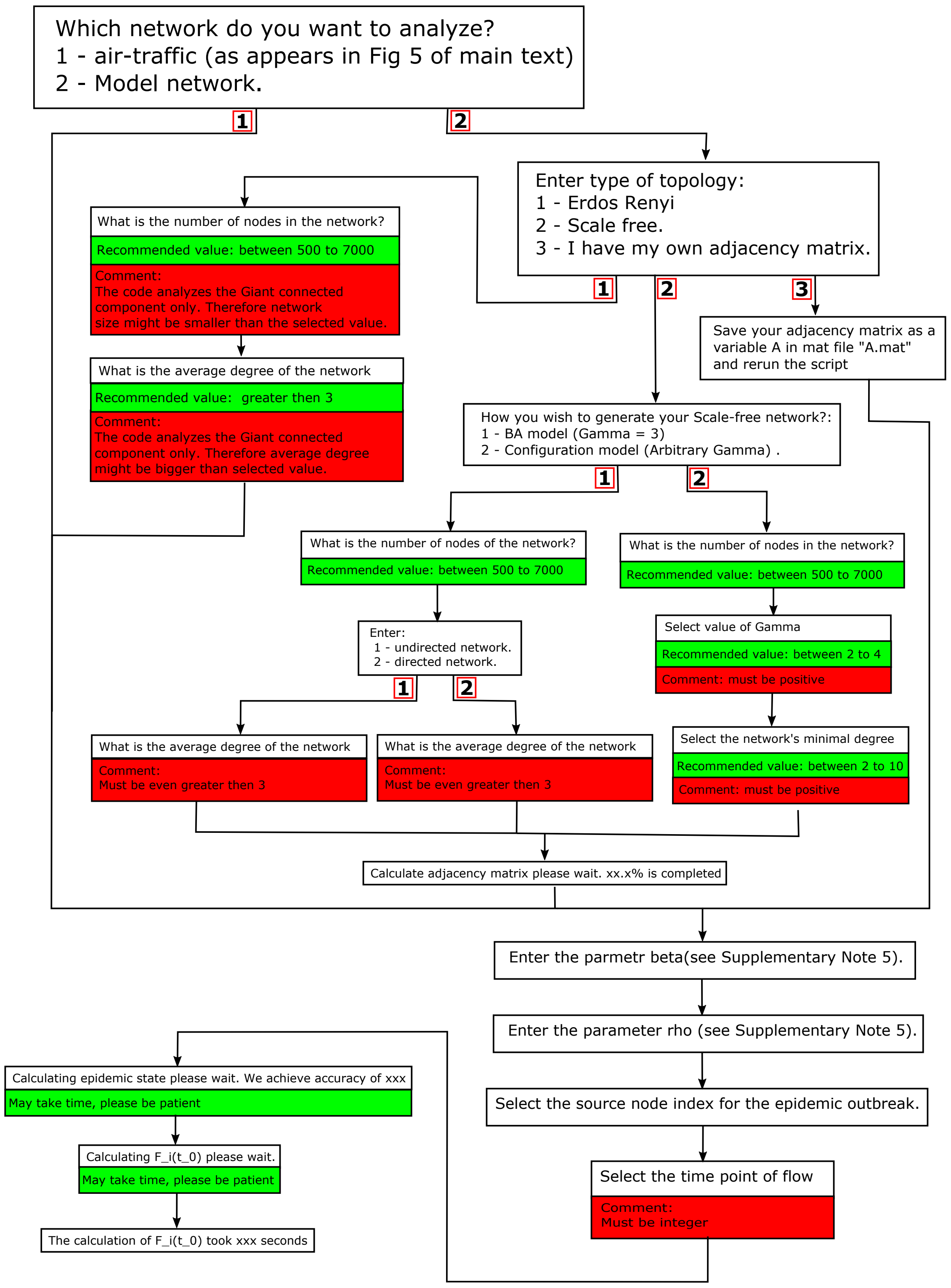

FIG. 2: **User interface for SIR model.**
